# Khoisan hunter-gatherers have been the largest population throughout most of modern-human demographic history

**DOI:** 10.1038/ncomms6692

**Published:** 2014-12-04

**Authors:** Hie Lim Kim, Aakrosh Ratan, George H. Perry, Alvaro Montenegro, Webb Miller, Stephan C. Schuster

**Affiliations:** 1 Center for Comparative Genomics and Bioinformatics, Pennsylvania State University, 310 Wartik Lab, University Park, Pennsylvania 16802, USA; 2 Singapore Centre on Environmental Life Sciences Engineering, Nanyang Technological University, 60 Nanyang Drive, SBS-01N-27, Singapore 637551, Singapore; 3 Department of Public Health Sciences and Center for Public Health Genomics, University of Virginia, Charlottesville, Virginia 22908, USA; 4 Departments of Anthropology and Biology, Pennsylvania State University, 513 Carpenter Building, University Park, Pennsylvania 16802, USA; 5 Department of Geography, Ohio State University, 154 North Oval Mall, Columbus, Ohio 43210, USA; 6 Campus do Litoral Paulista, Unesp—Univ Estadual Paulista, São Vicente 11330-900, Brazil

## Abstract

The Khoisan people from Southern Africa maintained ancient lifestyles as hunter-gatherers or pastoralists up to modern times, though little else is known about their early history. Here we infer early demographic histories of modern humans using whole-genome sequences of five Khoisan individuals and one Bantu speaker. Comparison with a 420 K SNP data set from worldwide individuals demonstrates that two of the Khoisan genomes from the Ju/’hoansi population contain exclusive Khoisan ancestry. Coalescent analysis shows that the Khoisan and their ancestors have been the largest populations since their split with the non-Khoisan population ~100–150 kyr ago. In contrast, the ancestors of the non-Khoisan groups, including Bantu-speakers and non-Africans, experienced population declines after the split and lost more than half of their genetic diversity. Paleoclimate records indicate that the precipitation in southern Africa increased ~80–100 kyr ago while west-central Africa became drier. We hypothesize that these climate differences might be related to the divergent-ancient histories among human populations.

## Abstract (web summary)

The expansion of Bantu agriculturalists 3,800 years ago in sub-Saharan Africa established first contact with Khoisan hunter-gatherers living in parts of Southern Africa. Sequencing the genomes of five Namibian-Khoisan hunter-gatherers and one Bantu individual tells a tale of admixture and isolation in the early history of modern human populations.

## Graphical abstract

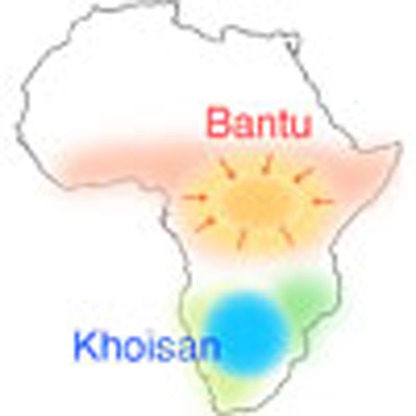

Following the rise of agriculture in sub-Saharan Africa ~4,000 years ago, Bantu-speaking subsistence agriculturalists spread rapidly throughout much of the sub-Saharan African continent^1^. Today, the census population sizes of these groups are orders of magnitude larger than those of sub-Saharan African hunter-gatherers, such as the Khoisan-speakers of the Kalahari Desert region in southern Africa^2^. Yet Khoisan populations have maintained the greatest nuclear-genetic diversity among all human populations^3^,^4^,^5^ and the most ancient Y-chromosome and mitochondrial DNA lineages^6^,^7^, implying relatively larger effective population sizes for ancestral Khoisan populations. While clues exist as to recent demographic histories (following the Bantu expansion) and interactions among sub-Saharan subsistence agricultural and hunter-gatherer groups, including evidence of admixture^8^,^9^, we know much less about the early (i.e., prior to the Bantu expansion) histories of these populations. In this study, we examine the early history of the ancestral hunter-gatherers and other human populations using analyses of complete-genome sequences from six individuals from southern Africa.

Previously, we reported the complete-genome sequences of a Namibian-Khoisan hunter-gatherer and a Bantu-speaking individual from Southern Africa, along with the exome sequences of three Namibian-Khoisan individuals^10^. In the current study, we sequence the complete genomes of five Namibian-Khoisan hunter-gatherers and one Bantu speaker, using the Illumina HiSeq platform to an average coverage of ~27–55-fold per individual (see details in Methods). We also include eight publicly available whole-genome sequences in our analysis (Table 1). Our analyses, using the genome sequences, reveal a larger effective population size for the ancestors of Khoisan following their split from non-Khoisan populations ~100–150 kyr ago, with a relatively dramatic population decline for the non-Khoisan populations. The divergent-population histories may be explained by concomitant-paleoclimate changes across Africa.

## Results

### Genetic origins of southern African individuals

**Figure 1:**
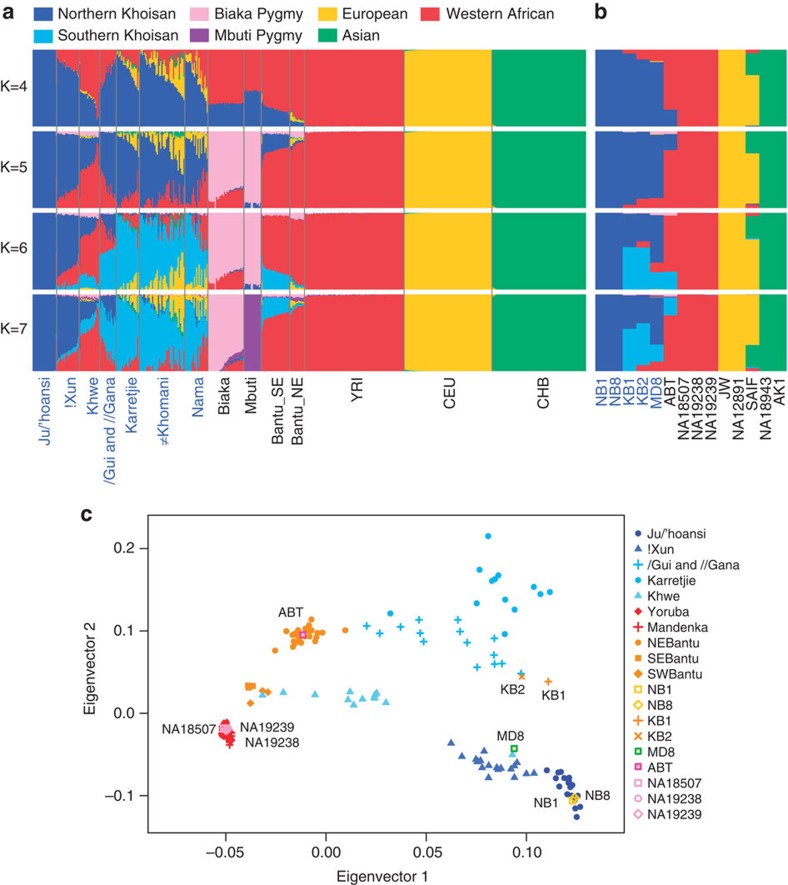
Genetic relationships of six southern African individuals and worldwide populations. (**a**) Population structure in human populations was inferred by *ADMIXTURE*^11^ using 417,593 SNPs from 490 individuals. (**b**) The *ADMIXTURE* plot for the 14 complete-genome data sets is shown separately. (**c**) Genetic relationships of our six southern African individuals and various African populations were estimated by the PCA analysis^12^ on the basis of the 417,593 SNPs from southern African and Yoruba populations. NB1 and NB8 are closely clustered with the Ju/’hoansi group, which was sampled from the northern Kalahari region in Namibia. The Ju/’hoansi samples are furthest from the Yoruba populations. MD8, from the northwestern Kalahari region, clusters with the !Xun, which belong to the same language group. KB1 and KB2, from the Tuu-speakers of the southern Kalahari, are close to the !Xun and /Gui and //Gana who lived in the central Kalahari region, but are not clearly related to them. Hence, we do not have any population data that is closely related to these two samples. ABT, a southern African Bantu, clusters with the southeastern Bantu samples.

In order to examine the genetic ancestries of the six individuals, we applied *ADMIXTURE*^11^ and EIGENSOFT^12^ to the genotyping data set of 419,969 nuclear single-nucleotide polymorphism (SNP) genotypes from 1,448 worldwide individuals along with genotypes extracted from the 14 genome sequences for the same SNP loci (Supplementary Table 1). Figure 1 shows the results for selected populations, emphasizing our six individuals. Entire results are shown in Supplementary Figs 1–3. On the basis of the *ADMIXTURE* result, Khoisan populations include two different ancestries, northern Khoisan and southern Khoisan, with evidence of past gene flow within the Khoisan and/or between the Khoisan and non-Khoisan, except for the Ju/’hoansi population (Fig. 1a). Individuals NB1 and NB8 belong to the Ju/’hoansi (Fig. 1c) and appear to have only northern Khoisan ancestry (Fig. 1b). We also applied a different method^13^, which uses linkage disequilibrium decay, to detect admixture between the Ju/’hoansi and other populations and show the result in Supplementary Fig. 7.

**Figure 2:**
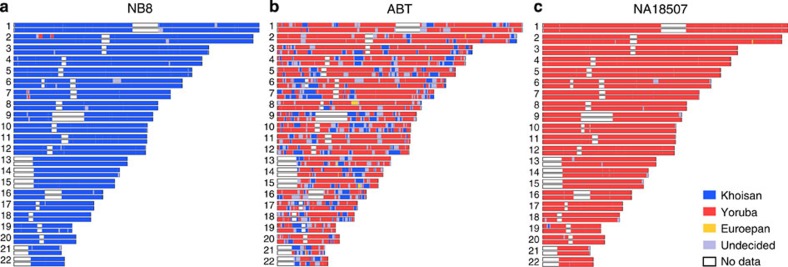
The local ancestry estimation for individual genomes. Along the genome, local ancestries are inferred by PCAdmix^40^ for NB8 (**a**), ABT (**b**) and NA18507 (**c**) and are illustrated on the genome map. Blue, red, yellow colors indicate the Khoisan (combined northern and southern Khoisan), western African and European ancestries, respectively. Light purple color represents undetermined ancestry that is not significant enough to estimate the ancestry. The western African haplotypes shown in the NB8 genome are not detected by the other two different methods (Supplementary Fig. 6 and Supplementary Table 2).

Inference of local ancestries along the genome using three-independent methods confirmed the exclusive Khoisan ancestry in the NB1 and NB8 genomes (Fig. 2, Supplementary Figs 4–7 and Supplementary Table 2). For the other Khoisan genomes—KB1, KB2 and MD8—the three methods and *ADMIXTURE* consistently assign 0.6–2.4% of each genome to western African ancestry (Supplementary Fig. 6 and Supplementary Table 2). ABT includes both western African and southern Khoisan ancestries, similar to the southeastern Bantu-speaking population (Fig. 1a). These results suggest a recent history of gene flow between the Khoisan and non-Khoisan populations, consistent with several other studies^3^,^5^,^14^,^15^,^16^, as well as, our previous report^10^ (Supplementary Fig. 8). However, we show here that two of the Ju/’hoansi genomes, NB1 and NB8, have no signature of admixture from non-Khoisan ancestries. Therefore their genome information allows us to access early population history of modern humans.

### Population-history inference

**Figure 3:**
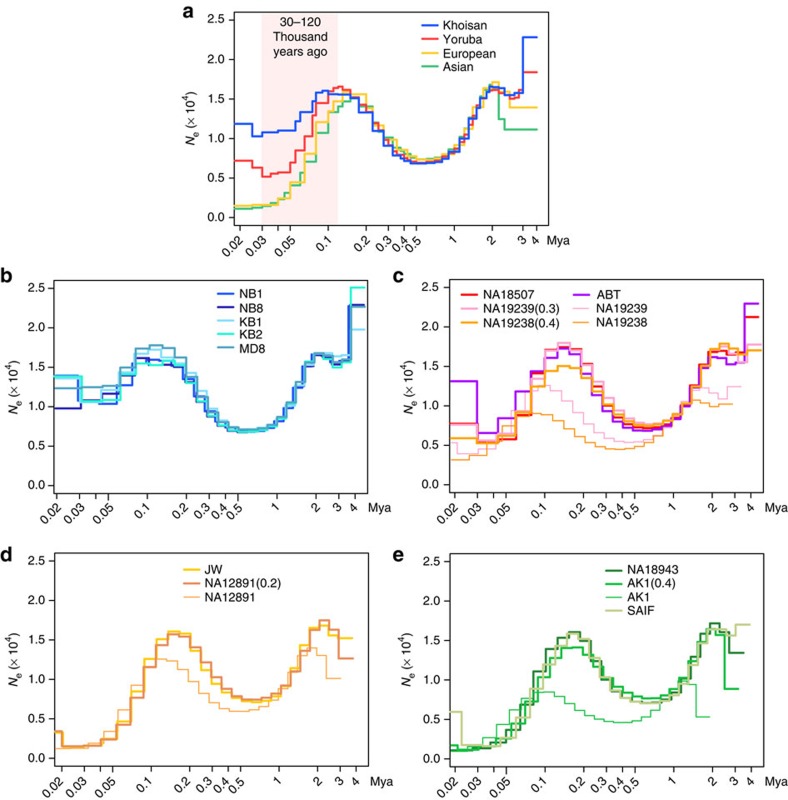
The changes in the effective population size on the basis of the 14 individual genomes. (**a**) The average *N*_e_ of each of four populations (see Methods). The pink shadow indicates the period where the changes in *N*_e_ varied most among the four populations. (**b**) *N*_e_ changes of five Khoisan genomes, (**c**) *N*_e_ changes of three Yoruba and one Bantu genome, (**d**) *N*_e_ changes of two European genomes, and (**e**) *N*_e_ changes of three Asian genomes. Four genomes sequenced to a relatively low coverage were corrected using the FNR option provided by the PSMC package. Estimates both with and without corrections are shown.

The Pairwise Sequentially Markovian Coalescent (PSMC) model^17^ was applied to the 14 whole-genome sequences in order to reconstruct the history of changes in effective population size (*N*_e_) over time. We used a typically reported mutation rate, 2.5e−08 per site per generation (generation time=25 years) (ref. 18), to scale *N*_e_ and time (see details in Methods). The patterns of change in *N*_e_ are consistent among the four populations (Khoisan, Yoruba, European and Asian) prior to ~0.2 myr ago, declining in all cases from 2 to 0.5 myr ago and recovering by 0.2 myr ago (Fig. 3a). All four populations appear to have experienced bottlenecks in the period ~30–120 kyr ago (Fig. 3a), but the declines in *N*_e_ varied widely among them (Fig. 3b–e). The Khoisan *N*_e_, the average of the two Ju/’hoansi genomes (NB1 and NB8), has been the largest since ~120 kyr ago and declined to 74% of their original peak *N*_e_ observed at about ~100–150 kyr ago, while the average *N*_e_ of the three Yoruba genomes declined to 31% of their original peak, followed by a slight recovery to 43%. The average *N*_e_ of each of two European and two Asian genomes declined even more, to only 9 and 8% of their original peak, respectively (Fig. 3a).

We performed simulations to assess the robustness of these PSMC results under various demographic models. Genome sequences were generated by simulations under a simplified model of the population size changes inferred by PSMC from the Khoisan and Yoruba genomes. PSMC was applied to the simulated sequences, and we confirmed that the PSMC inference reconstructs the given model (Supplementary Fig. 9). Several reports have found evidence of recent admixtures between the Khoisan and non-Khoisan populations^15^, a population structure within the Khoisan^5^,^15^ and the Bantu population expansion within Africa^1^. Since the PSMC only estimates changes of effective population size and does not account for population structure, we used these simulations to examine effects of recent demographic events on the PSMC estimates. The PSMC estimates from the sequences simulated under the models including recent demographic events are not significantly different from the estimate from the sequence simulated under the model without those events (Supplementary Fig. 9). These simulations demonstrate that the large Khoisan *N*_e_ and Yoruba population decline that we estimated from the Ju/’hoansi and Yoruba genomes are not a result of the recent demographic events.

In addition, we could infer the divergence time of populations from the PSMC analysis, using male X chromosomes^17^. The earliest human population split has been known to be between the ancestral Khoisan and the ancestors of the other human populations and was estimated to take place ~110–150 kyr ago (refs 16, 19). Our PSMC analysis and a Bayesian inference^19^ support similar estimates, ~120–150 kyr ago (Supplementary Fig. 10) and ~95–130 kyr ago (Supplementary Fig. 11), respectively.

On the basis of these results, we can reconstruct early history of modern-human populations. After the earliest split, between the ancestral Khoisan and non-Khoisan populations ~100–150 kyr ago, the ancestral Khoisan population maintained their high genetic diversity, while the effective population size of the non-Khoisan continued to decline for 30~120 kyr ago and lost more than half of its diversity. The ‘Out of Africa’ migration ~40–60 kyr ago (ref. 20) accounts for the observed population split between African and non-African populations, and the subsequent smaller effective population size of non-Africans compared with non-Khoisan Africans.

### Climatic changes in Africa

**Figure 4:**
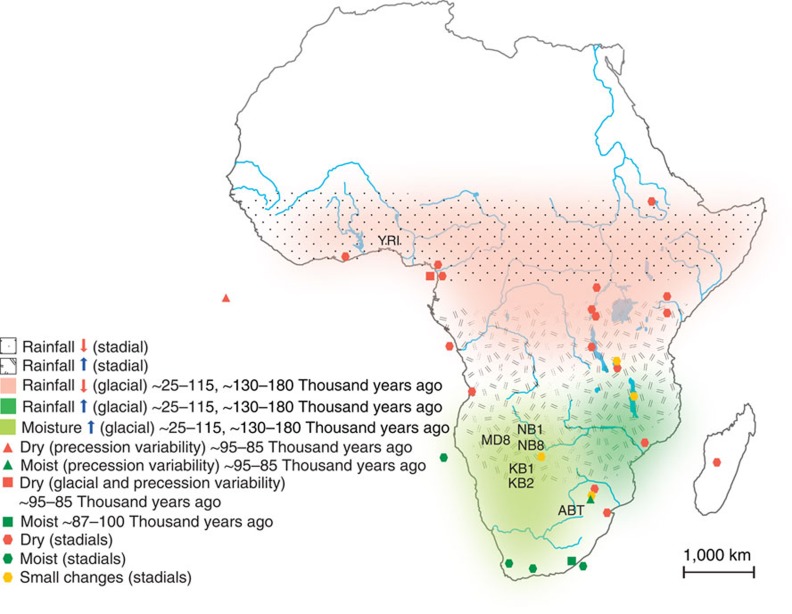
The climate changes in the African continent. Modes of African rainfall variability characterized by opposite changes in precipitation along the north–south axis. With the exception of the light green area over southwestern Africa, colors and patterns refer to modelled results. Symbols refer to proxy records. Local conditions during particular periods are given by dates in front of some legend entries. Stadials have millennial time scale and were recorded several times around ~100 kyr ago. The names of our six southern African and Yoruba (YRI) individuals refer to their sampling location. References for this figure are indicated in Supplementary Table 3. The original map was retrieved from d-maps.com and edited by authors.

We focused on environmental changes during the time period of the dramatic decline in effective population size observed in our analysis of the Yoruba genomes, compared with the Khoisan. Climate changes may have impacted populations in west-central Africa, contemporaneous with environmental conditions that did not change or even improved for populations in southern Africa. Paleoclimate records and numerical models point to three modes of African precipitation variability (each with distinct causes, temporal and spatial scales) that fit such a pattern (Fig. 4).

First, there is ample proxy evidence that much of Africa tends to be drier under glacial conditions^21^,^22^. Climate models show this to be due to mostly colder north Atlantic waters, stronger northern-hemisphere trade winds and weaker summer monsoons^22^. The exception, registered in many climate archives, are the wetter conditions found over southwest Africa during the glacial ~25–115 kyr, believed to be brought about by an increase in winter storm activity in the region^23^. Analysis of oceanic sediment show a significant increase in moisture over southwest Africa between ~100–120 kyr ago, the initial stages of the last glacial^24^.

Second, precession of the Earth’s axis of rotation generates a ~23 kyr cycle in summer insolation. Models and proxy-data show that this affects monsoon intensity leading to changes in African rainfalls that are out of phase between the hemispheres^25^. Oceanic and lacustrine archives indicate that the period between ~87–94 kyr ago was marked by increased precipitation in southern Africa accompanied by drier conditions over the central, western and eastern portions of the continent, and that these changes correlate to variations in summer insolation caused by precession^26^,^27^.

Third, stadials—millennial time scale events characterized by cooling in the northern-hemisphere high latitudes—have also been related in both models and proxies to rainfall increases in southern Africa accompanied by drying over central and western areas^28^,^29^. A particularly wet period recorded in the southern tip of the continent at ~91 kyr ago has been associated with this mode of precipitation variability^29^.

## Discussion

On the basis of the parallel found between climate changes with the divergent-population history within Africa, we propose the following hypothesis regarding early human history. Modern humans may have originated anywhere in Africa and spread across the continent, with continuous gene flow among the populations. From ~100–150 kyr ago, the human species was geographically structured within Africa and eventually differentiated genetically owing to limited gene flow. At or after the time of the population differentiation, a drier climate began to affect the western and central, but not the southern regions of the African continent. This potentially contributed to a relatively severe decline in the western African populations (ancestors of the current Bantu-speaking populations) while the size of southern African populations, ancestors of the current Khoisan, was maintained or declined to a much lesser degree. Non-Africans, the majority of modern humans alive on the planet today, represent a subpopulation split from the ancestral non-Khoisan African population^3^,^8^,^15^, and their genetic diversity further dramatically decreased during their migration from Africa to Eurasia (Supplementary Fig. 12).

As described in the Methods, a neutral mutation rate of 2.5e−08 was used to scale the time axis in our PSMC analysis. However, recent studies have reported a lower mutation rate^30^,^31^,^32^ than the one we used. If we use the lower mutation rate, the population-history differentiation starts about 100 kyr earlier, ~200–250 kyr ago (Supplementary Fig. 13). Our hypothesis regarding early human history still holds with this lower mutation rate, as this would put the population-history divergence at or after the time close to the initial stages of the next-to-last glacial ~200 kyr ago (Fig. 4) (refs 22, 24), when an important determinant of precipitation would be changing in a way similar to what happened ~100 kyr ago.

Our hypothesis may explain the distinctive demographic histories among human populations and suggest that the Khoisan hunter-gatherers and their ancestors have been the largest population in terms of genetic diversity throughout modern-human history. This is in stark contrast to the current census size of the Khoisan hunter-gatherers, which is today drastically smaller compared with that of the Bantu-speaking populations. Further research into the population structure of the Ju/’hoansi and related Khoisan groups with larger sample size, therefore, will be essential for a comprehensive understanding of the deep divergence and population history of modern humans.

## Methods

### Southern African DNA sampling

A study permit was obtained from the Ministry of Health and Social Services (MoHSS), Namibia. Ethics approval to conduct whole-genome sequencing and analysis was obtained from the Institutional Review Board (IRB) or Human Research Ethics Committee (HREC) from three institutions, namely the Pennsylvania State University (IRB #28460 and IRB #28890), the University of New South Wales, Australia (HREC #08089 and HREC #08244) and the University of Limpopo, South Africa (Limpopo Provincial Government #011/2008).

Consents of the participants were obtained verbally (and documented via videotape) or in writing. The consent text was provided in English, Afrikaans or via an interpreter in the native language of each participant (Juu- and Tuu-language). Participants agreed that the data generated will be made freely available to the scientific community.

We consented three males and two females from two indigenous hunter-gatherer groups in the Northern and Southern Kalahari Desert. Each was among the eldest members of their respective communities. Inclusion in the study was also on the basis of their narrated-family history, as well as the remoteness of their geographical location, impeding ease of contact with other groups.

The indigenous Kalahari hunter-gatherers included in this study live in scattered family groups in the vast semi-desert regions of Namibia, an 823,145-km^2^ country on the southwest coast of Africa with ~2 million inhabitants (ref. 10 and references therein). Today Namibia is home to ~38,000 Khoisan people. In detail, KB1 and KB2 are members of a Tuu-speaking group of the southern Kalahari. NB1 and NB8 are Ju/’hoansi of the northern Kalahari region, separated by ~600 km aerial distance. MD8, belongs to the !Xun (!Kung)-speaking group relocated by the government from the Etosha plains region in the northwestern Kalahari. ABT is a direct descendant from the two major-linguistic groups in southern Africa, namely the Nguni-speakers (~60% of the people of South Africa) via his paternal Xhosa ancestry and from the Sotho-Tswana-speakers (~33% of the people of South Africa) via his maternal Motswana ancestry.

### Genome sequencing and read alignments

The samples NB1, NB8, KB1, KB2, MD8 and ABT were sequenced to a depth of 27–55-fold using the Illumina HiSeq sequencing platform. The details regarding samplings, DNA extraction and sequencing are the same as described in Schuster *et al.*^10^ The genome sequences for the eight other human samples were downloaded from the NCBI Short Read Archive (SRA). These sequences were aligned to the human reference sequence (GRCh37/hg19) using the BWA (version 0.5.9) aligner (ref. 33). All default parameters were used with the exception of ‘–q 15’, which was used to soft-trim the low quality bases at the 3′ ends of the reads. The reads were then realigned using the GATK IndelRealigner (ref. 34), and the potential PCR duplicates were flagged using the MarkDuplicates tool from the Picard suite (Picard, http://picard.sourceforge.net). Sequence-read data for the six southern African genomes that were sequenced as part of this study have been deposited in the Sequence Read Archive under accession PRJNA263627.

### SNP and consensus calls

The diploid consensus sequence for the autosomes was obtained using the ‘mpileup’ command. The option ‘–C 50’ was used to reduce the mapping quality of the reads with multiple mismatches. Locations were marked as missing data in the following cases: (a) The coverage at the location was less than three reads or greater than twice the average coverage of the genome; (b) The RMS mapping quality of the reads at the location was less than 10. The consensus for the X chromosome was derived similarly, but the pseudo-autosomal regions (chrX:60001-2699520 and chrX:154931044-155260560) were filtered as missing data. The heterozygous calls in the male X chromosomes were also discarded as errors.

We used SAMtools version 0.1.18 (ref. 33) to identify the locations of the SNPs, using the option ‘–C 50’ to reduce the mapping quality of the reads with multiple mismatches. SNP locations in the nuclear genome were filtered to maintain SNPs for which coverage in the sample was less than that expected using the Lander–Waterman equation^35^. We filtered out variant locations where the RMS mapping quality was less than 10, or if the SNP quality was less than 30.

### Genotyping data sets

We obtained three genotyping SNP data sets from the following sources: genotyping data from HapMap^36^, HGDP (CEPH, http://www.cephb.fr/en/hgdp), and Schlebusch *et al.*^5^ (Supplementary Table 1). We identified the SNPs common to the three data sets. We merged those with the SNPs from our 14 whole-genome data sets. The resulting data set was then filtered to throw away flipped SNPs (SNPs on the non-reference strand) or possible flipped SNPs: the AT-GC SNPs, the triple-allelic SNPs within the merged data sets, as well as the SNPs that were homozygotes within each data set. This left us with 419,969 SNPs.

For this study, we selected only unrelated individuals. Five pairs of individuals, three Ju/’hoansi and two Southwestern Bantu, were identified as highly related, according to the Identical By Descent (IBD) analysis, which was run using PLINK^37^. The following five individuals were removed from the data sets based on the greatest fraction of missing data: KSP113, KSP116, KSP117, KSP196, and KSP205. Moreover, for analyses in which we identified populations, we removed the genetic-outlier individuals with respect to their populations. To detect outlier individuals, we performed PCA on the merged genotyping data set. We identified two individuals for removal as outliers in their population: HGDP00980 and KSP109. Therefore, we used 419,969 SNPs from 1,462 individuals for our population genetic analyses in this study (Supplementary Table 1). This SNP data set is available on ‘Bushman’ data library on Galaxy^38^.

### Population structure and admixture estimations

We applied the *ADMIXTURE* program^11^ to the merged SNP data set to identify ancient population structure and ancestries of the 14 complete-genome samples. To reduce the effects of biased SNPs on clustering groups in the program, we used only 417,593 SNPs having a minor-allele frequency greater than 0.01 in the entire population of the 1,462 individuals. First, we used the entire data set of 1,462 individuals for the analysis with the number of ancestries *K*=4–14 (Supplementary Fig. 1). The higher *K* distinguished many isolated ethnic groups in Asia. Thus we ran the program using selected 490 individuals which are composed of African, European and Asian populations (Fig. 1).

Independently, we performed PCA analysis of the SNP genotyping data set, using EIGENSOFT^12^. We applied the analysis to the entire data set of 1,462 individuals (Supplementary Fig. 2) and then to only African and European populations including Central Asians (*n*=967) to examine the gene flow between African and European ancestries (Supplementary Fig. 3). To identify origins of our six southern African individuals precisely, we performed the PCA analysis for only African populations (*n*=274; Fig. 1c). In this analysis, the ≠Khomani and Nama populations were not included because of their recent gene flow from non-Khoisan ancestries, in order to better examine genetic clusters of individuals.

In order to identify admixtures in each of the six southern African individuals’ genome, we applied three methods: HAPMIX^39^, PCAdmix^40^ and dpmix^41^. To prepare the data sets for the run of PCAdmix, we needed to determine phased genotypes. The phased genotype data set of the same HapMap panel ( http://bochet.gcc.biostat.washington.edu/beagle/1000_Genomes.phase1_release_v3/) and the data set by Schulebusch *et al.*^5^ For the unphased data sets, we inferred a haplotype phase using BEAGLE^42^. PCAdmix requires three ancestor populations in order to infer the ancestry of each haplotype. Based upon the *ADMIXTURE* results, the samples that contained a high proportion (>0.8) of the corresponding ancestry were selected as the ancestral population, because the genetic components of the ancestral populations would likely affect the inference by PCAdmix. We used the Khoisan (*n*=67), Yoruba (*n*=85) and European (*n*=82) populations as putative ancestral populations for inference of ancestry of our Khoisan individuals. For the inference of ABT, we used a southeastern Bantu population with ABT as a test population. For the inference of non-African genomes, we assumed Yoruba, European and Asian (*n*=81) ancestral populations. We fixed the window size of the haplotype to 40 SNPs and used default for other parameters in the PCAdmix analysis. The estimated local ancestry of our six southern African samples and each from Yoruba, European and Asian samples were illustrated in Supplementary Fig. 4.

Since HAPMIX assumes only two ancestral populations, we ran the program for two sets of ancestral populations (Khoisan/Yoruba and Khoisan/European) independently. Two parameters of lambda and theta were given: the time of admixture and the admixed proportion, respectively. For both runs, we fixed lambda=5, and theta as 0.01 for admixtures from the Yoruba population and 0.001 for admixtures from the European population, according to the results of the *ADMIXTURE* analysis, which showed no significant admixtures from European ancestry. Before we determined the parameters, we tested several sets of parameters, and the results were pretty robust. The two sets of outputs (Khoisan/Yoruba and Khoisan/European) were unified into a single result set. When inconsistent estimates occurred between the two outputs, such as KHO/KHO versus KHO/YRI, the admixed type (KHO/YRI) was chosen, so that the unified result was biased toward detecting admixtures. When two different admixtures were inferred for one genotype, for example, KHO/YRI versus KHO/EUR, it was classified into the ‘undetermined’ category.

The third method of inferring admixtures was applied, by using the dpmix program^41^ on Galaxy^38^. We used the ‘Admixture’ tool in the ‘Genome diversity’ session. The only parameter we needed to determine was ‘genotype switch penalty’: we fixed the parameter of 10, which determined a reasonable length of admixture blocks, after testing genome switch penalties in the 2–20 range. The dpmix program allowed us to assume three ancestral populations, and we used the same three ancestral populations defined for the PCAdmix analysis.

We compared results between those three methods and collected the SNPs for which all three methods supported a consistent ancestry. The SNPs showed inconsistent ancestry among methods were assigned into the ‘undetermined’ category. The consistent results were illustrated in Supplementary Fig. 5, and a comparison between methods was shown in Supplementary Fig. 6 and Supplementary Table 2.

### Effective population size inference

We ran the PSMC program^17^ on each of our 14 whole-genome sequencing data sets in order to infer effective population size. For the run, we used the consensus called using SAMtools as described before. The parameters and options used with PSMC were the same as the ones used in a previous study^17^. We measured the variance of the estimate by bootstrapping, using the option provided in the PSMC package. We repeated the run 100 times for each of the individuals (Supplementary Fig. 14).

There were a couple of issues relative to the understanding of the PSMC estimates. Since PSMC uses the heterozygote density for its inference, sequencing quality affects the PSMC estimates significantly. Sequence coverage of the 14 genome data sets varied between 19–86-fold (Table 1). Considering the associations of sequencing coverage and number of heterozygous sites (Supplementary Fig. 15), the ABT genome was sequenced to a high enough coverage (27-fold) to identify the heterozygotes. In addition, we designed an experiment to examine the impact of sequencing coverage on the PSMC estimates. We generated eight genome data sets having 10–80-fold sequencing coverage from one Yoruba genome (NA18507), which, at 86-fold, has the highest sequencing coverage, among our data sets (Table 1). Using the eight data sets, we ran PSMC independently. The pattern of changes in the PSMC estimates was very similar among those genome data sets: however the degree of change was shifted toward being more recent and smaller in size as the sequencing coverage was lowered (Supplementary Fig. 16). From this experiment, we found that a sequencing coverage cutoff of 30-fold average is sufficient for robust PSMC outputs. Therefore, half of our complete-genome sequencing data sets are suitably qualified for PSMC analysis (Table 1).

Only four genome data sets used in this study were not sequenced to a higher coverage than the ABT genome: NA12891, NA19238, NA19239 and AK1. The PSMC estimates of these four genomes were corrected, using the ‘false negative rate (FNR)’ option provided by the PSMC package. We used the KB1 genome data set to test the effectiveness of this option. We generated the KB1 genome sequence, having 15-fold sequencing coverage, and ran PSMC (KB1.15X). We used the FNR option to correct the estimates, which were shown to be KB1.15X (FNR=0.1) and KB1.15X (FNR=0.2), respectively in Supplementary Fig. 17. The original KB1 estimate was pretty similar to KB1.15X (FNR=0.2). We ended up using the FNR option for the four genomes NA12891, NA19238, NA19239 and AK1 to adjust the effect of low sequencing coverage on the PSMC estimates (Fig. 3).

The second issue was that scaling the estimates depends upon the mutation rate. Estimates of both effective population size (*N*_e_) and time depend upon the mutation rate assumed in the PSMC analysis. Classically, the mutation rate has been known to be 2.5e−08 per site per generation^18^, while recent studies reported a lower rate of 1.2e−08 per site per generation^30^,^31^,^32^. In this study, we used 2.5e−08 per site per generation as a mutation rate, because the mutation rate estimated by a Bayesian inference (G-PhoCS; Supplementary Fig. 11) was around 2.5e−08, on the basis of our genome data set. The effect of the mutation rate is described in Supplementary Fig. 12.

In addition, the PSMC estimates for the very recent time period, such as 20–42 kyr ago to present, may not produce accurate estimations, according to the author^17^ and our bootstrap test (Supplementary Fig. 14). Therefore, we don’t discuss PSMC estimates in relation to recent history ~20 kyr ago, in this study.

### Coalescence simulations

In order to confirm a robustness of the PSMC inference (Fig. 3), we performed coalescence simulations. Under several demographic models, nucleotide sequences were generated, using the *ms* program^43^. Commands used in this analysis and details are shown in Supplementary Fig. 9. The PSMC program was applied to the simulated sequences to confirm to reconstruct the effective population-size over time as given model and effects of recent demographic events on the PSMC estimations of past effective population size.

### The estimation of population divergence time

We used PSMC analysis to infer the divergence time between populations^17^,^44^. If PSMC analysis is applied, a hybridized diploid of two haploid sequences from each population, then the time point where the inferred population size increased corresponds to the divergence time. To apply this approach, we constructed each pseudo-diploid X chromosome by combining two male X chromosomes. Supplementary Fig. 10 shows the results of PSMC analysis for pairwise pseudo-diploid X chromosomes. For example, the pairs of the European genome (JW) and each of the Khoisan, Yoruba and Asian populations are shown in Supplementary Fig. 10a. The Khoisan and European pair (JW-NB1) demonstrated a clear increase in their population size compared with the European pair (JW-NA12891) from 150–313 kyr ago, which could be the divergence time of the Khoisan and non-Khoisan populations. All other pairs stayed low in population size relative to the Khoisan and European pair, until 270–570 kyr ago. This result clearly shows the earliest population splits between the Khoisan and non-Khoisan populations, even though the time estimation is approximate.

We also applied G-PhoCS^19^ to our genome data set to estimate divergence time. We assumed the simplest model, illustrated in Supplementary Fig. 11, to estimate the divergence time of the Khoisan and non-Khoisan population. Both the NB1 and NB8 genomes were used as the Khoisan genome and were applied independently. The NA18507 genome was used as the western African genome. In total, randomly selected 20,000 loci of 1–2 kb-length were used as input data for the G-PhoCS run to reduce computational requirements. To prepare the input data, we followed the filtering procedure described in Gronau *et al.*^19^ In order to confirm a robustness of outputs, various sets of parameters (models, priors) were applied to the G-PhoCS run independently. We carried on 100,000 iterations for the burn-in period and an additional 200,000 iterations for the sample collection. The acceptance rates for all parameters ranged 20–70%. The analysis for the samples was performed using Tracer v1.5 (ref. 45). The estimates from the run were pretty robust and are shown in the table in Supplementary Fig. 11. The estimated mutation rate was around 1.0e−9 per site per generation, the same as 2.5e−8 per site per generation, if generation time is assumed to be 25 years. The estimates of divergence time were calibrated by the human and chimpanzee divergence time, 5.6–7.6 myr ago^46^. The mutation rate was calculated by an estimate of *τ*_div_=6.5e−03, and the human-chimpanzee divergence time (*T*_div_) as *μ*=*τ*_div_/*T*_div_.

## Author contributions

S.C.S. collected samples and generated data and designed the study; H.L.K. and G.H.P. were involved in study design; A.R. and W.M. performed bioinformatics analyses; A.M. analyzed paleoclimate records and literatures; H.L.K. performed population genetic analyses and drafted the manuscript; All authors participated to write the paper.

## Additional information

**Accession codes:** Whole-genome sequencing data generated in this study have been deposited in GenBank/EMBL/DDBJ sequence-read archive (SRA) under the accession code PRJNA263627.

**How to cite this article**: Kim, H. L. *et al.* Khoisan hunter-gatherers have been the largest population throughout most of modern-human demographic history. *Nat. Commun.* 5:5692 doi: 10.1038/ncomms6692 (2014).

## Supplementary Material

Supplementary Information Supplementary Figures 1-17, Supplementary Tables 1-3, Supplementary Methods and Supplementary References

## Figures and Tables

**Table 1:** The 14 complete-genome sequencing data sets.

**Sample**	**Sex**	**Population**	**Mt lineage**	**Reference covered (%)**	**Coverage**	**Number of SNP calls**	**Heterozygote calls**
NB1	M	Ju/’hoansi	L0k1a	99.9	30	4,378,009	2,599,257
NB8	F	Ju/’hoansi	L0d1c1b	99.3	43	4,527,368	2,778,246
KB1	M	Tuu-speaker	L0d1b2	99.9	55	4,531,748	2,757,367
KB2	F	Tuu-speaker	L0k1a	99.3	29	4,443,595	2,756,268
MD8	M	!Xun	L0k1a	99.9	41	4,478,374	2,777,909
ABT	M	South African Bantu	L0d2a’b	99.9	27	4,151,778	2,627,717
NA18507	M	Yoruba	L1b1a3	99.9	86	4,247,930	2,711,171
NA19238	F	Yoruba	L3e2b	99.4	19	2,760,241	1,493,532
NA19239	M	Yoruba	L2a1	99.9	23	3,142,138	1,772,904
JW	M	European	U8a1a1	99.9	39	3,374,491	1,996,657
NA12891	M	European	H1e1a	99.9	26	2,808,162	1,553,776
SAIF	F	Southern Indian	U1a3	99.2	33	3,490,568	2,096,023
NA18943	M	Japanese	M7b8	99.9	31	3,386,469	1,859,833
AK1	M	Korean	D4g2a1	99.9	21	2,336,316	1,010,456
