# Supplementary material for: Khoisan hunter-gatherers have been the largest population throughout most of modern-human demographic history
Source: Nat Commun. 2014 Dec 4;5:5692. doi: 10.1038/ncomms6692 (PMC4268704; doi:10.1038/ncomms6692)
Supplement: Supplementary Information — Supplementary Figures 1-17, Supplementary Tables 1-3, Supplementary Methods and Supplementary References [file ncomms6692-s1.pdf]

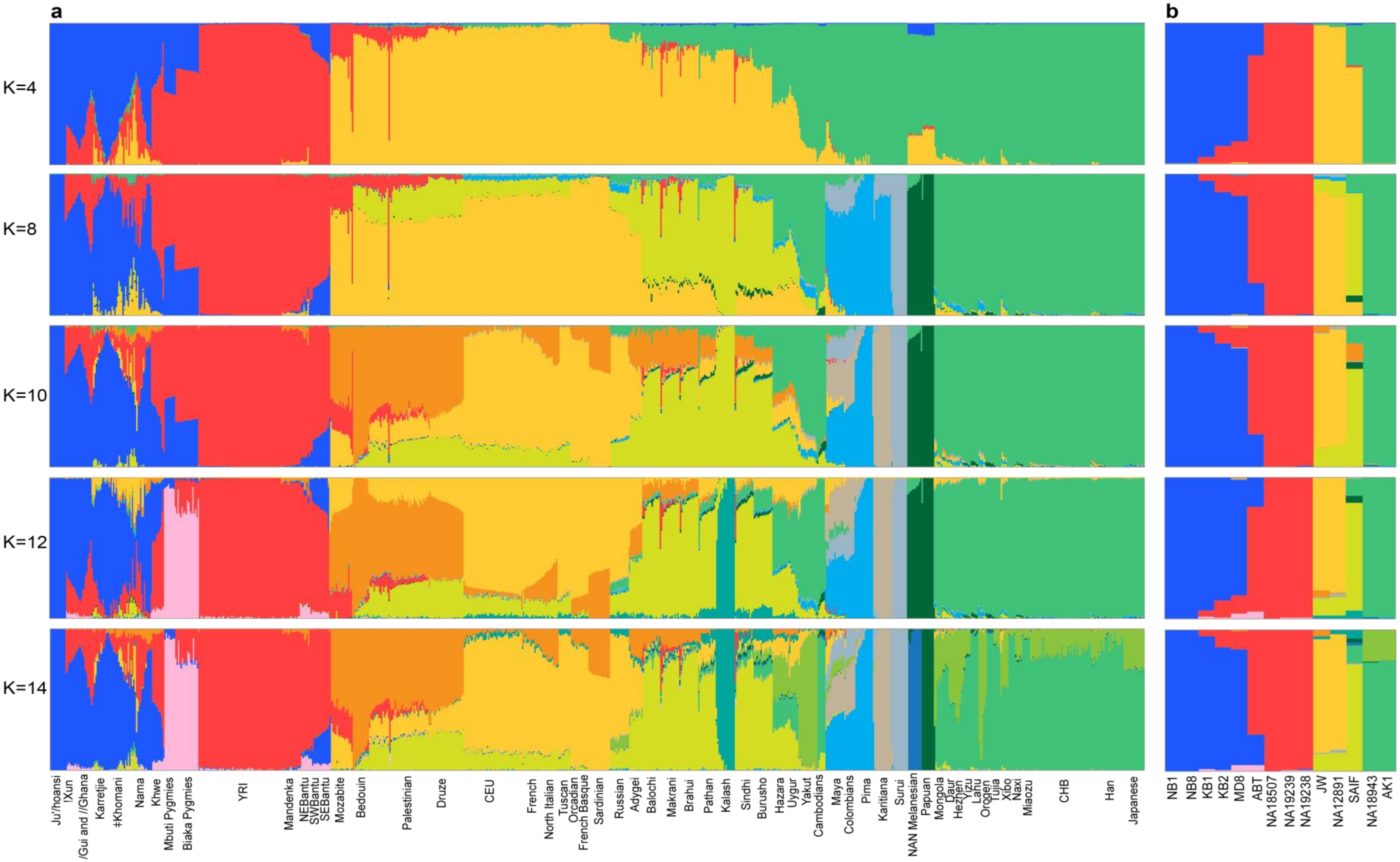

Supplementary Figure 1. Population structure for worldwide populations.
The population structure estimated by *ADMIXTURE* (*11*) using the SNP genotyping dataset, 417,593 SNPs (minor allele frequency > 0.01) from 1,448 individuals, is illustrated in the bar graph (**a**). The 14 individuals from the complete genome dataset are shown separately (**b**).

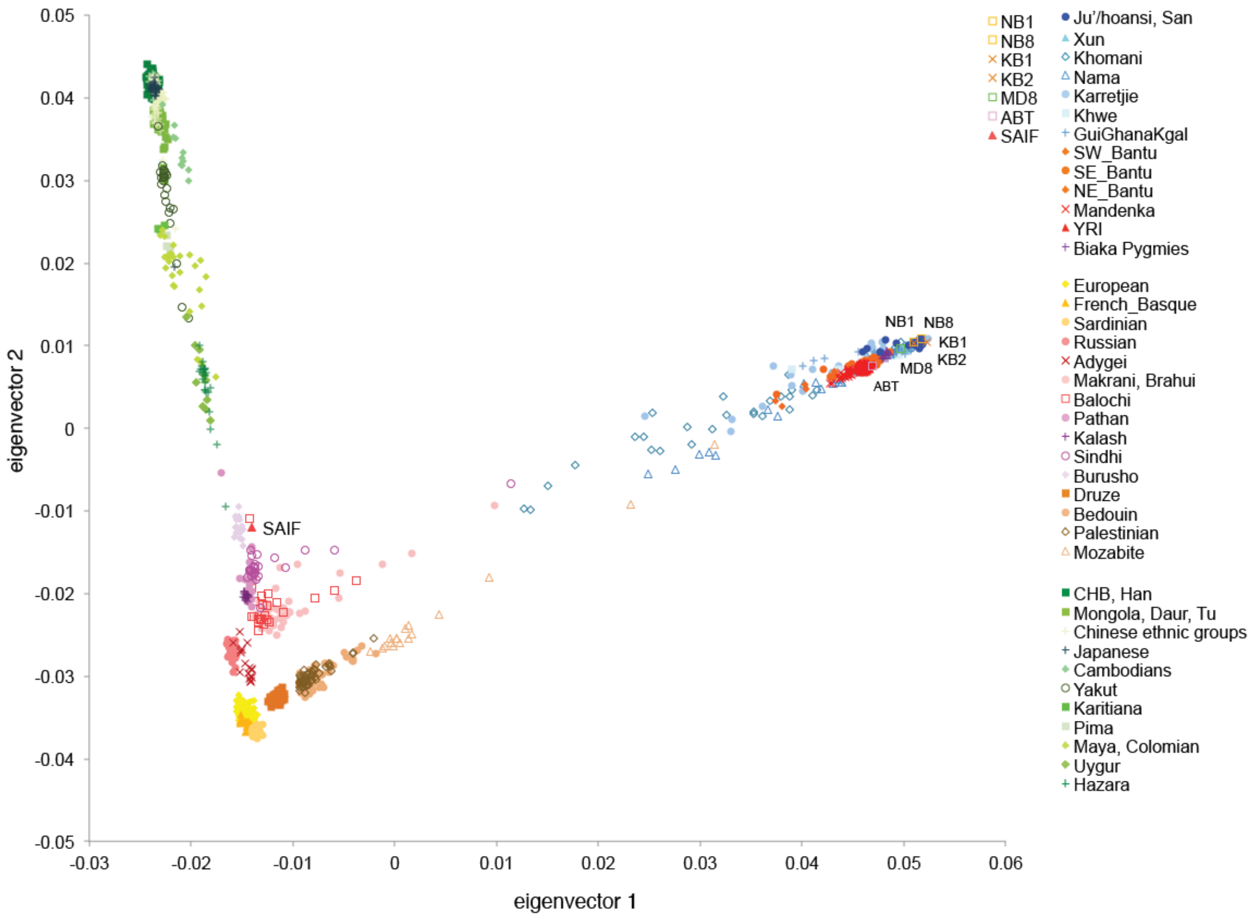

Supplementary Figure 2. PCA plot for worldwide populations.

The principal component analysis (PCA) was performed on the 417,593 SNPs from 1,462 individuals from worldwide populations, by using EIGENSOFT (12). There are three main populations, Africans, Europeans, and Asians. Many populations suggest gene flow between Europeans and Asians and between Europeans and Africans.

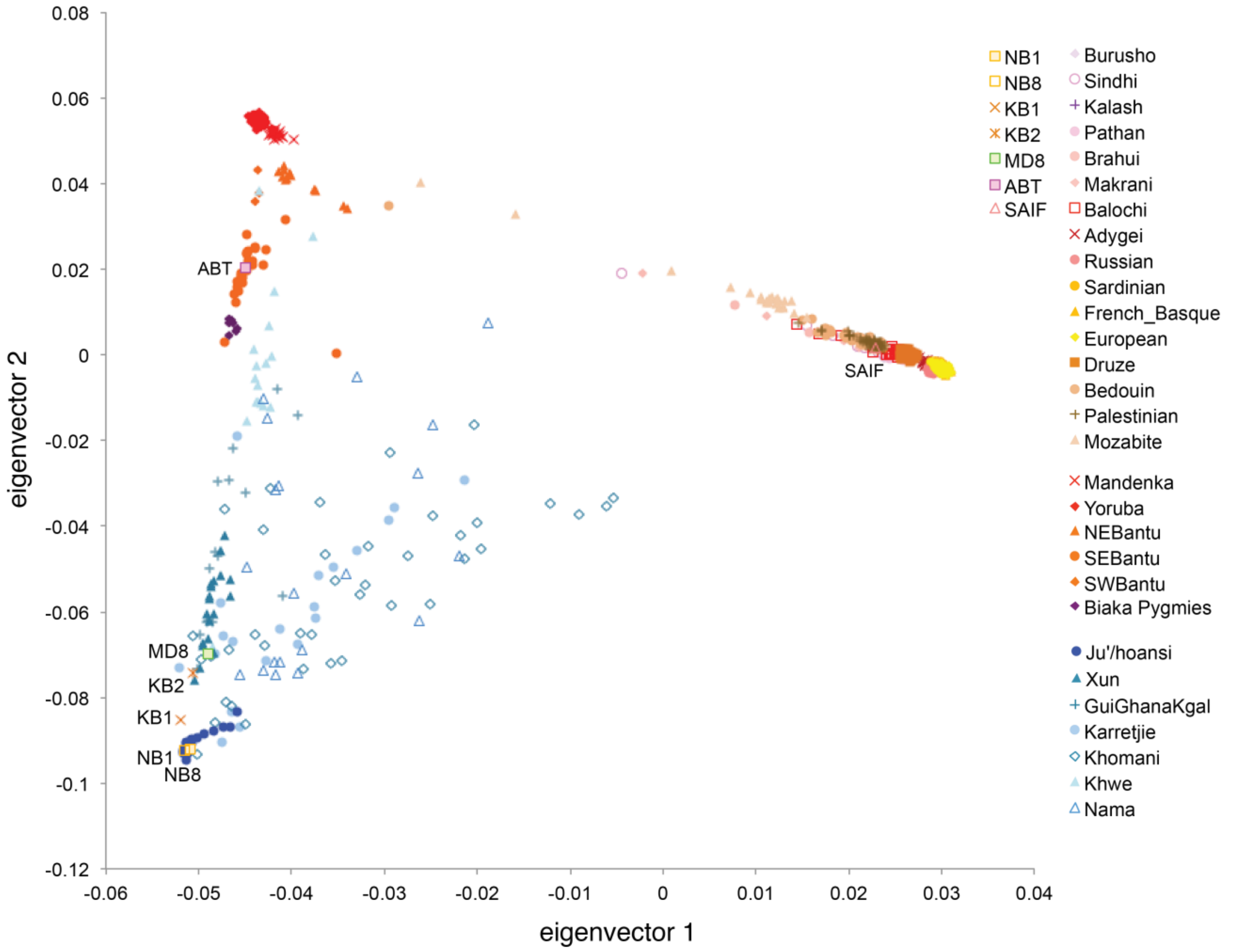

Supplementary Figure 3. PCA plot for African and European populations. To examine gene flow between Africans and Europeans, we performed the PCA analysis for 967 selected individuals from African and European populations, including central Asians, based on the analysis using the entire populations (Supplementary Fig. 2). In this plot, three clusters suggest three diverged populations containing each of Khoisan, Yoruba (Bantu), and European ancestries. Many Khoisan populations, except for Ju/'hoansi, suggest admixture from Bantu and European ancestries.

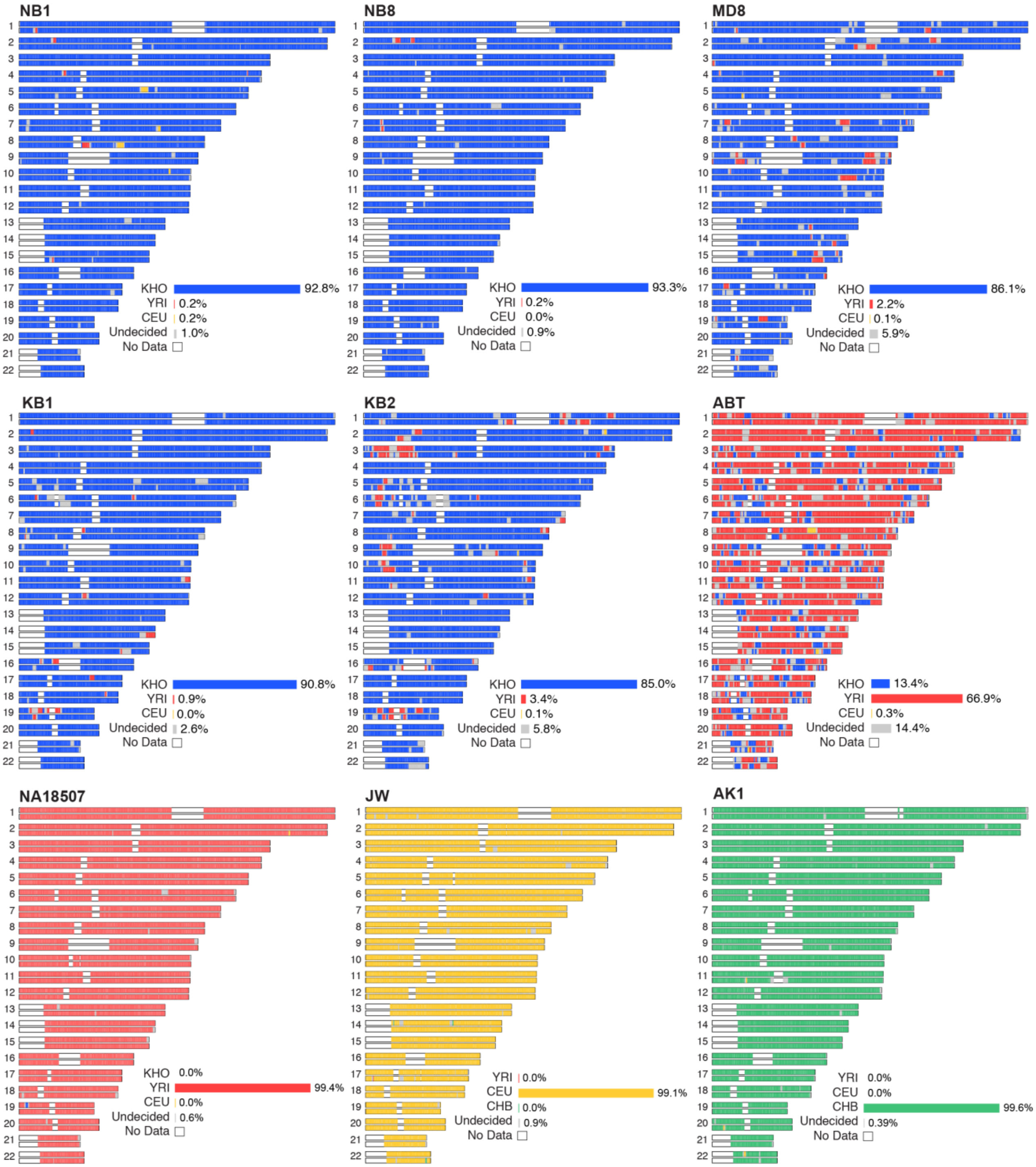

Supplementary Figure 4. Local ancestry assignments along chromosomes. Estimated ancestry was assigned to each SNP along chromosomes of the six southern African and three non-African genomes, using PCAdmix (41). 420K SNPs in 67 Khoisan (KHO, blue), 85 Yoruba (YRI, red), 82 European (CEU, yellow), and 81 Asian (CHB, green) individuals were used as putative ancestral populations. The color of each haplotype block represents the assigned ancestry. The length of the color bars in the right bottom of the chromosome map indicates the proportion of each ancestry in the entire genome.

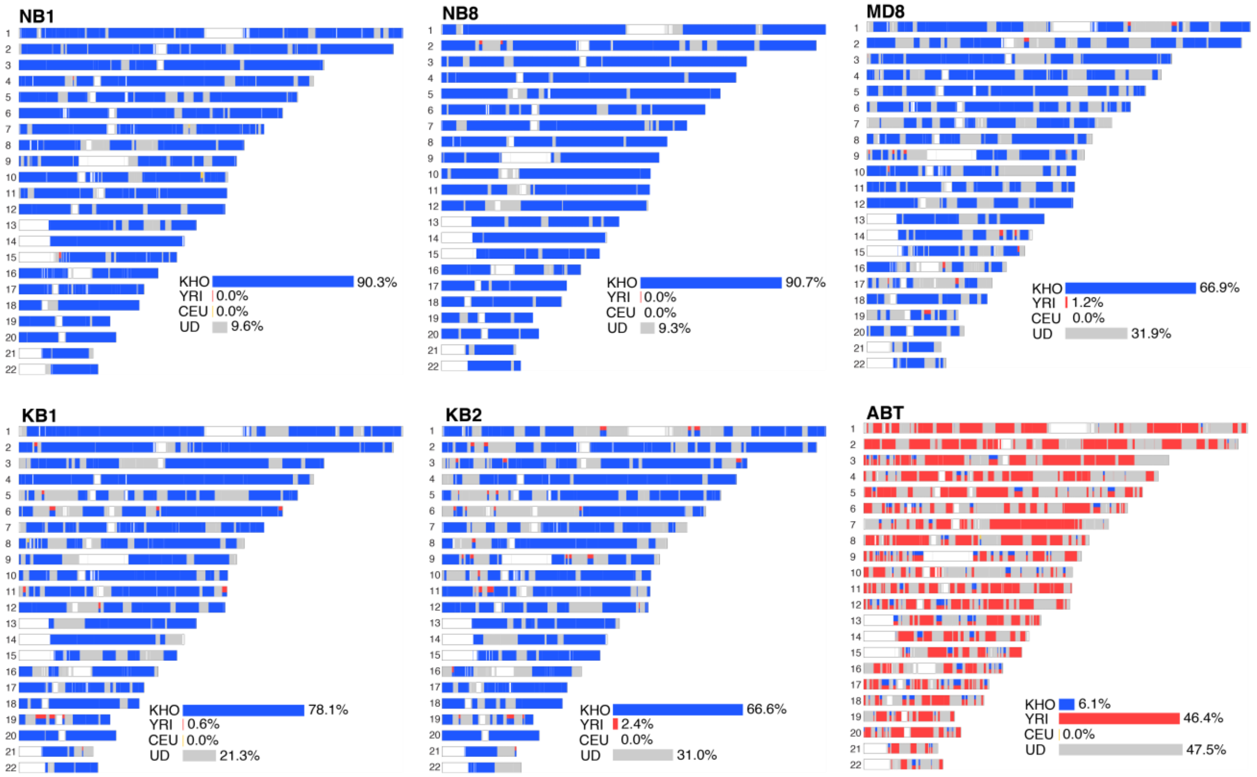

Supplementary Figure 5. Local ancestry estimation consistently supported by three methods. The chromosome map illustrates the results; a blue bar indicates a SNP that was identified as a Khoisan (KHO) SNP or located in the Khoisan haplotypes: red and yellow bars represent a western Africa (YRI) and European (CEU) SNP, respectively. The SNPs for which consistent ancestry could not be determined among the methods (undetermined, UD) are shown as gray bar. The proportion of each ancestry identified by each method is shown in Supplementary Figure 6 and Supplementary Table 2.

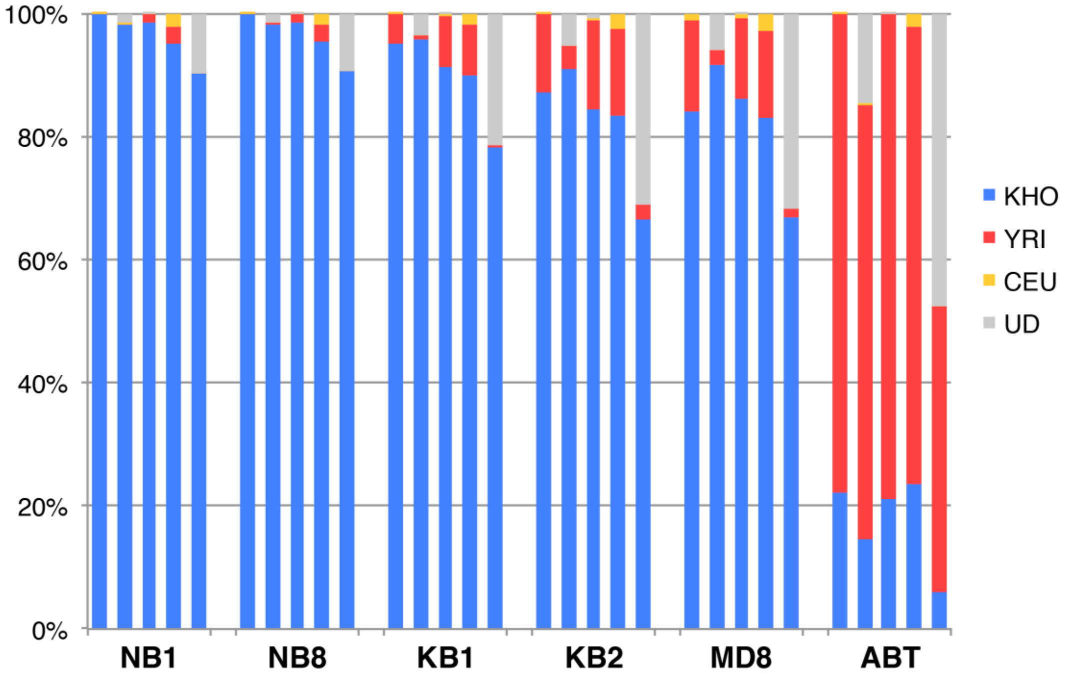

Supplementary Figure 6. Comparison of admixture estimations among four methods. Based on Supplementary Table 2, the proportions of ancestries were graphed for each individual. Within an individual, the five bars illustrate each result of *ADMIXTURE*, PCAdmix, HAPMIX, dpmix, and consistent results among the three methods in order of left to right.

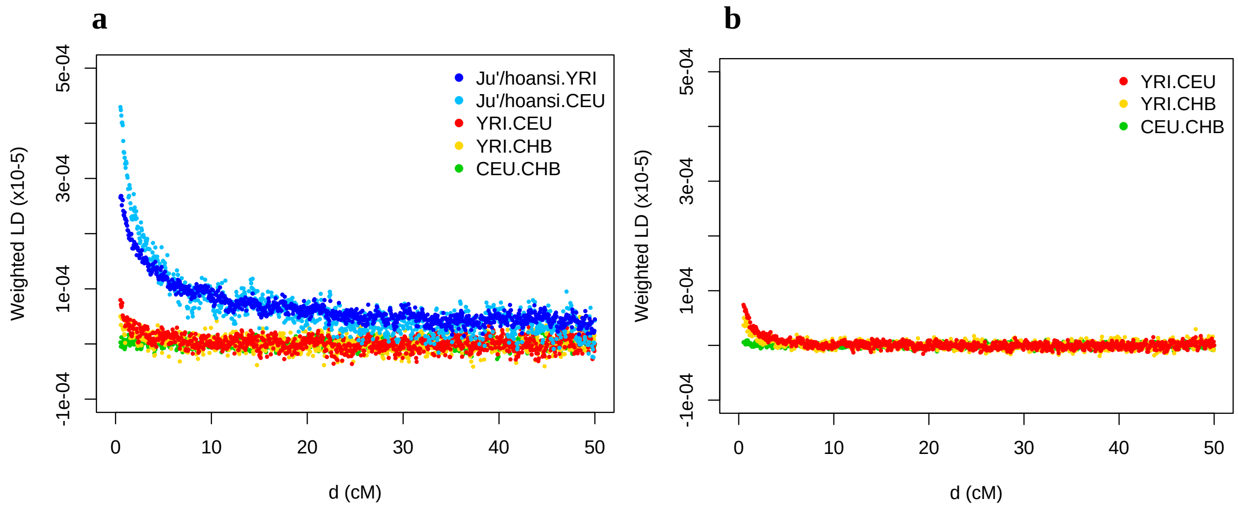

Supplementary Figure 7. ALDER analysis for detecting admixture between Khoisan and non-Khoisan populations.

A method for detection of admixture between diverged populations based on Linkage Disequilibirum (LD), ALDER (13) was applied to our five Khoisan individuals (NB1, NB8, KB1, KB2, and MD8) and the Ju/'hoansi population (n=19), using the 417,593 SNP genotyping dataset. In the LD decay plot, the X and Y axis indicate genetic distance and weighted LD between a pair SNPs, respectively. The correlation of LD decay between the test and reference populations supports occurrence of admixture between populations. **a**) The LD decay plot for the five Khoisan individuals as a test population. The admixture test provided in the program was not successfully performed, due to too strong relationships between the test and Ju/'hoansi population as shown in the plot, and inconsistent LD decay rates between the test and reference populations. The five samples as a test population may not be sufficient to apply the statistical test. Also we certainly know three genomes among the five samples have admixtures. Therefore as a test sample of five genomes, we cannot distinguish the potential admixture of two Ju/'hoansi samples from this analysis. **b**) The LD decay plot for the Ju/'hoansi population related with YRI, CEU, and CHB populations. The YRI.CEU reference population shows a significant correlation with the Ju/'hoansi population. The tests with other reference populations are not successful due to the same reason mentioned before. At least we can infer that there is correlation between the Ju/'hoansi and YRI populations but a few correlation between the Ju/'hoansi and CEU.CHB populations based on the LD decay plot. For the five Khoisan individuals, the YRI ancestry found in KB2 and MD8 in the previous analyses could be detected as the slight relationship with the YRI.CEU reference. The admixture test based on LD decay has limitations in application to our dataset and to identify local ancestries for each individual genome.

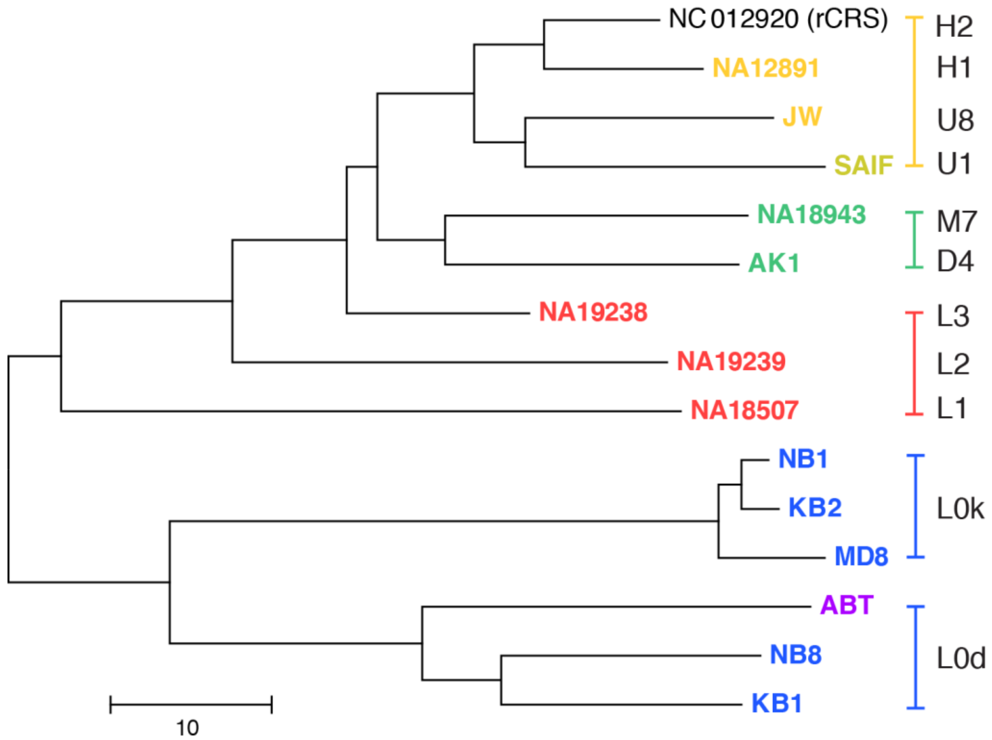

Supplementary Figure 8. Phylogenetic relationships of the mitochondrial genome of the 14 individuals. The neighbor-joining tree was constructed based on the number of different nucleotides in the complete mitochondrial genome of the 14 individuals and the reference sequence (NC012920), using MEGA5 (50). The mitochondrial haplotype was identified by HaploGrep (51). The ABT mitochondrial genome belongs to the L0d haplotype which is specific to the Khoisan population, suggesting admixture from Khoisan to Bantu.

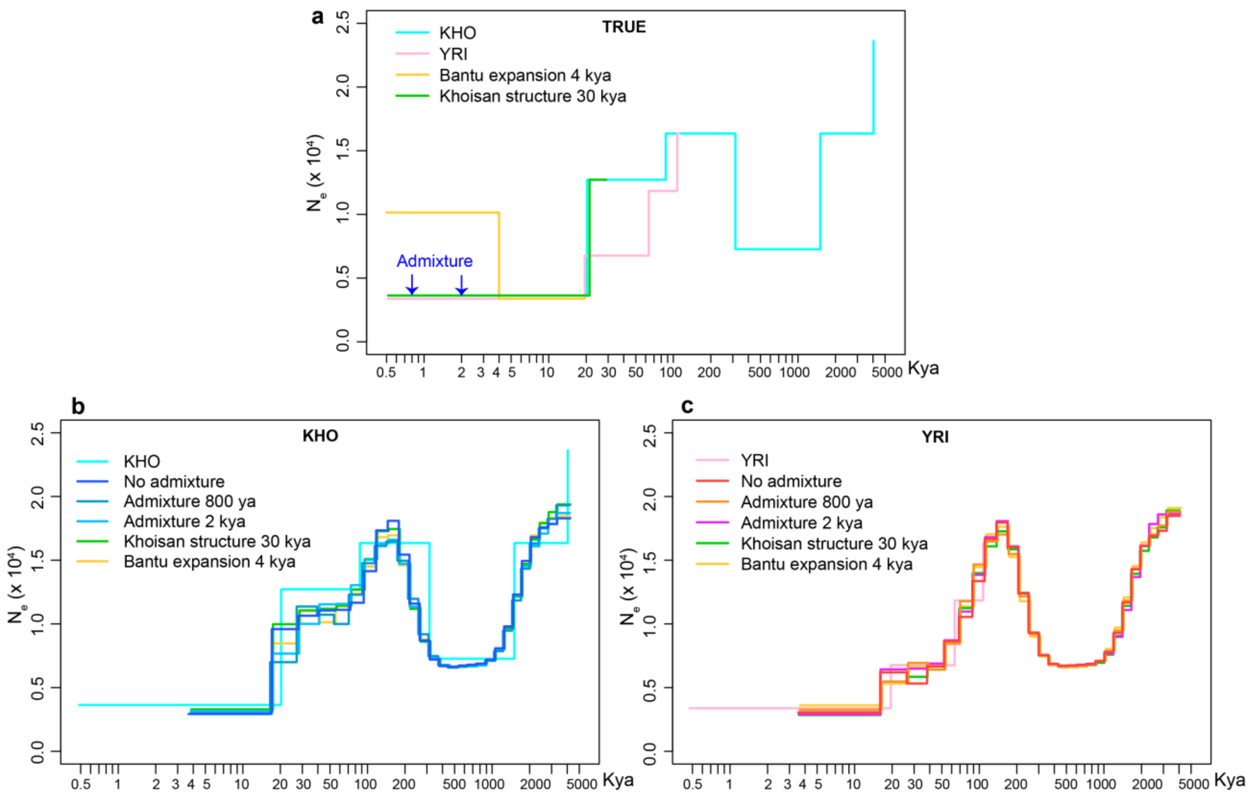

Supplementary Figure 9. The PSMC inference from simulated sequences.
In order to confirm effects of recent demographic events on the PSMC estimates from the Khoisan genomes used in this study (Fig. 3), coalescence simulations were performed using *ms* program (*46*). **a**) Based on the given *ms* commands generated by the 'history2ms.pl' script included in the PSMC package, we combined the Khoisan and Yoruba population history and simplified the demographic model, as shown in the plot (KHO, YRI). In addition, we introduced admixture between the two populations 800 ya according to the previous study (*15*) with two amounts of migration rates (Nm = 1, 4). We separately assumed the Bantu population expansion 4 kya (*1*) and the population structure within the Khoisan (northern and southern Khoisan) occurred 30 kya (5, *15*). **b**) The PSMC inference for the simulated Khoisan sequences for each demographic model. The inference well reproduced the given demographic model. Compared to the estimate without any recent demographic events (blue), those estimates assumed admixture (sky blue), population structure (green), and population expansion (yellow) are not significantly different from each other. Independently, we tested another model, which assumes that the Khoisan also experienced the same level of bottlenecks as the Yoruba, and induced the same series of recent demographic events as same as this model. The results are consistent with that the recent events did not impact to the PSMC inference. **c**) The PSMC inference for the simulated Yoruba sequences for each demographic model. As like the Khoisan (**b**), the recent demographic events did not impact to the PSMC estimations. The used *ms* commands are below.

<ms command>

*KHO&YRI: ./ms 4 100 -t 10560 -r 1833 30000000 -p 7 -I 2 2 2 -ej 0.2783 2 1 -en 0.0507 1 3.5 -en 0.2172 1 4.5 -eN 0.7865 2.0 -eN 3.7725 4.5 -eN 10.1067 6.5 -en 0.0488 2 2.0 -en 0.1589 2 3.5 -en 0.2685 2 4.5 -eM 0 0

*Admixture (*Nm*=1): ./ms 4 100 -t 10560 -r 1833 30000000 -p 7 -I 2 2 2 -ej 0.2783 2 1 -en 0.0507 1 3.5 -en 0.2172 1 4.5 -eN 0.7865 2.0 -eN 3.7725 4.5 -eN 10.1067 6.5 -en 0.0488 2 2.0 -en 0.1589 2 3.5 -en 0.2685 2 4.5 -eM 0 1 -eM 0.002 0

*Admixture (*Nm*=4): ./ms 4 100 -t 10560 -r 1833 30000000 -p 7 -I 2 2 2 -ej 0.2783 2 1 -en 0.0507 1 3.5 -en 0.2172 1 4.5 -eN 0.7865 2.0 -eN 3.7725 4.5 -eN 10.1067 6.5 -en 0.0488 2 2.0 -en 0.1589 2 3.5 -en 0.2685 2 4.5 -eM 0 4 -eM 0.002 0

*Khoisan structure 30 kya: ./ms 6 100 -t 10560 -r 1833 30000000 -p 7 -I 3 2 2 2 -ej 0.075 3 1 -em 0 1 3 1 -em 0 3 1 1 -em 0 1 2 0 -em 0 2 1 0 -ej 0.2783 2 1 -en 0.0507 1 3.5 -en 0.0507 3 3.5 -en 0.2172 1 4.5 -eN 0.7865 2.0 -eN 3.7725 4.5 -eN 10.1067 6.5 -en 0.0488 2 2 -en 0.1589 2 3.5 -en 0.2685 2 4.5

*Bantu expansion 4 kya: ./ms 4 100 -t 10560 -r 1833 30000000 -p 7 -I 2 2 2 -ej 0.2783 2 1 -en 0.0507 1 3.5 -en 0.2172 1 4.5 -eN 0.7865 2.0 -eN 3.7725 4.5 -eN 10.1067 6.5 -en 0 2 3 -en 0.01 2 1.0 -en 0.0488 2 2 -en 0.1589 2 3.5 -en 0.2685 2 4.5 -eM 0 0

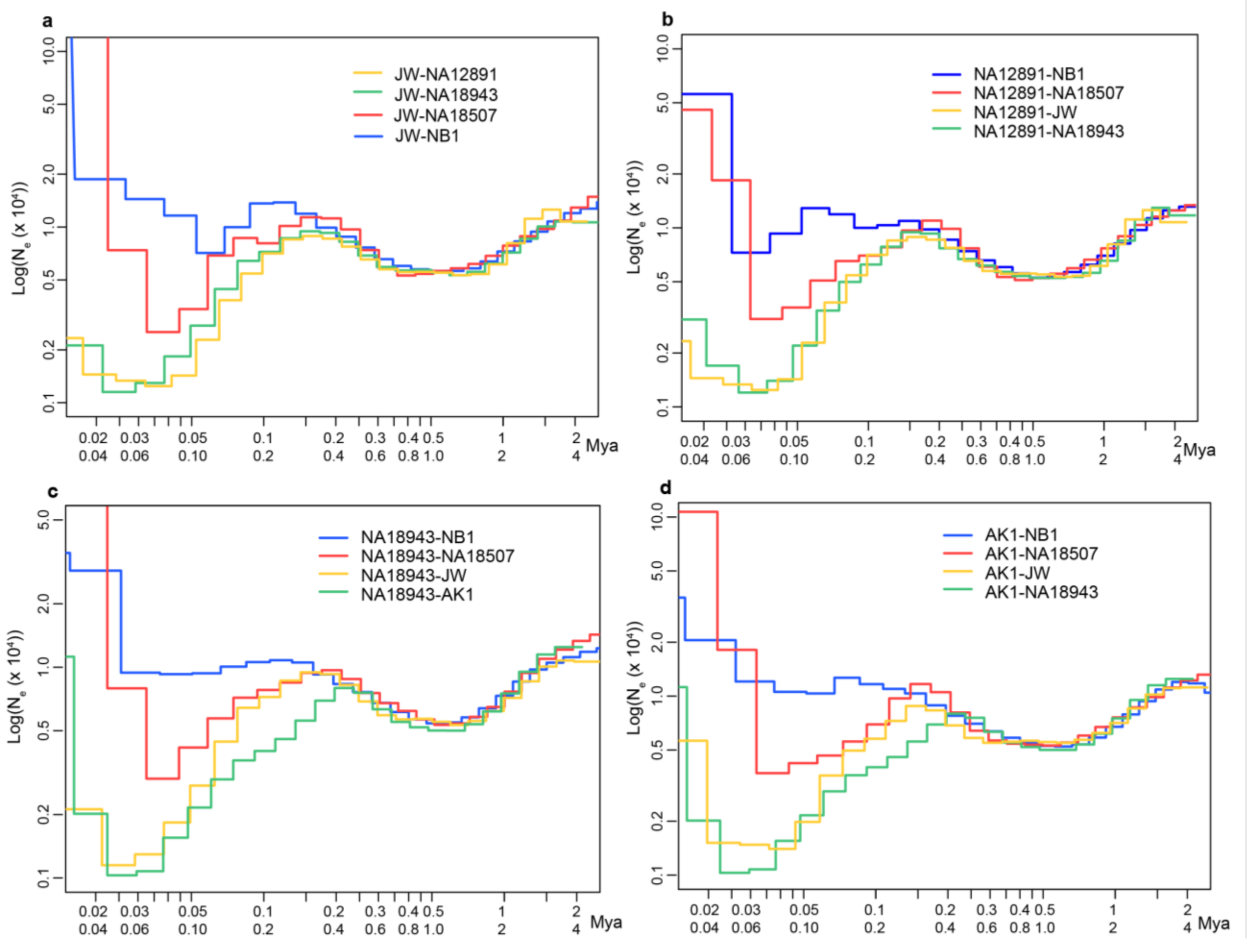

Supplementary Figure 10. Estimations of divergence time using male X chromosomes. The time was adjusted using parameters of a generation time (25 years) and two mutation rates (2.5e-08/site/generation and 1.2e-08/site/generation) and is indicated along the X axis. The Y axis indicates the log-scaled effective population size. Each line shows the population size inferred from a pair of two male X chromosomes. The time point of the split between Khoisan and Yoruba X chromosomes is around 150 Kya based on 2.5e-08/site/generation mutation rate.

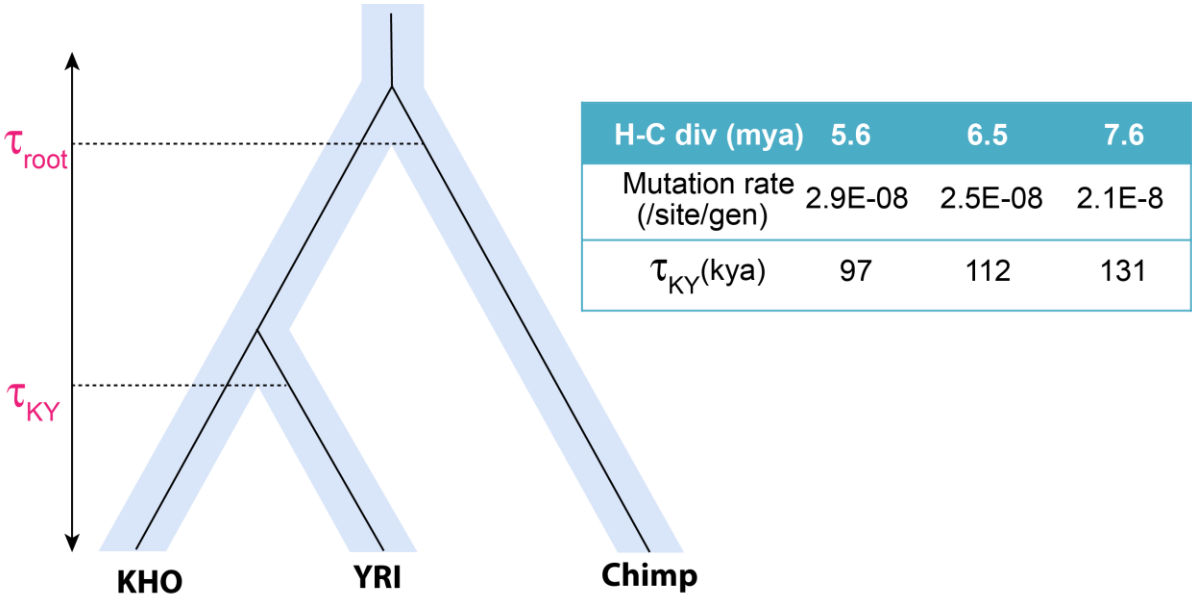

H-C div (mya)	5.6	6.5	7.6
Mutation rate (/site/gen)	2.9E-08	2.5E-08	2.1E-8
τ_{KY}(kya)	97	112	131

Supplementary Figure 11. Bayesian inference of the Khoisan and western African population divergence time.

Under the assumption of the demographic model, the population split time (τ) was estimated using G-PhoCS (19). The estimates are calibrated by the human and chimpanzee divergence time, 5.6~7.6 mya (49). The mutation rate was calculated by an estimate of τ_{div} = 6.5e-03, and the human-chimpanzee divergence time (T_{div}) as $\mu = \tau_{div} / T_{div}$.

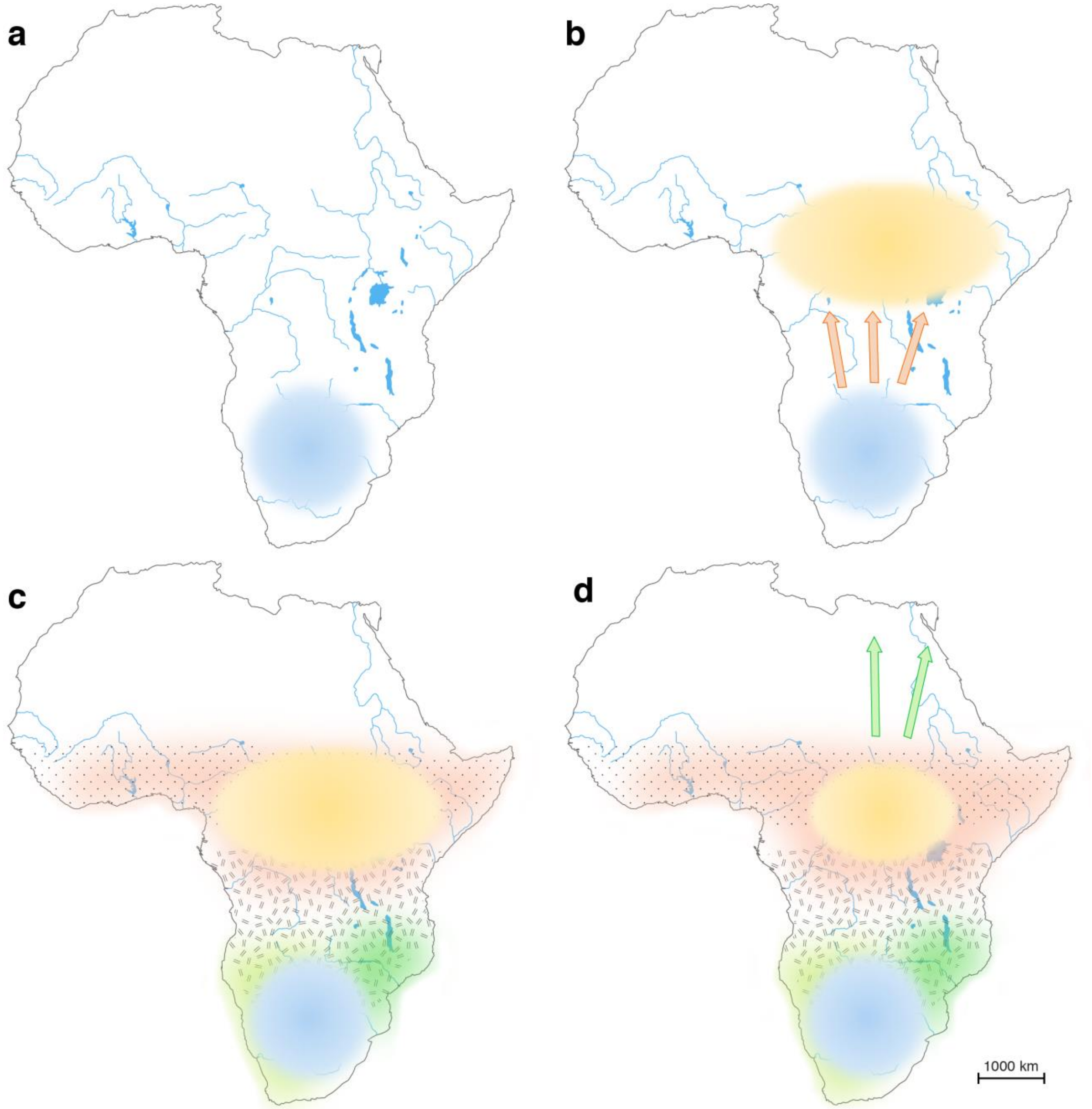

Supplementary Figure 12. The hypothesis for the human population history within Africa. The place and size of populations in the figure are hypothetical. **(a)** Modern humans may have originated anywhere in Africa. **(b)** The population spread across the continent. **(c)** From ~100-150 kya, the human species was geographically structured within Africa and eventually differentiated genetically due to limited gene flow. At or after the time of the population differentiation, a drier climate began to affect the western and central, but not the southern regions of the African continent. **(d)** This potentially contributed to a relatively severe decline in the western African populations (ancestors of the current Bantu-speaking populations) and/or maintained the size of southern African populations, ancestors of the current Khoisan. The non-Africans, the majority of modern humans alive on the planet today, are a subpopulation split from the ancestral Bantu (*3, 8, 15*), and their genetic diversity dramatically decreased during their migration from Africa to Eurasia.

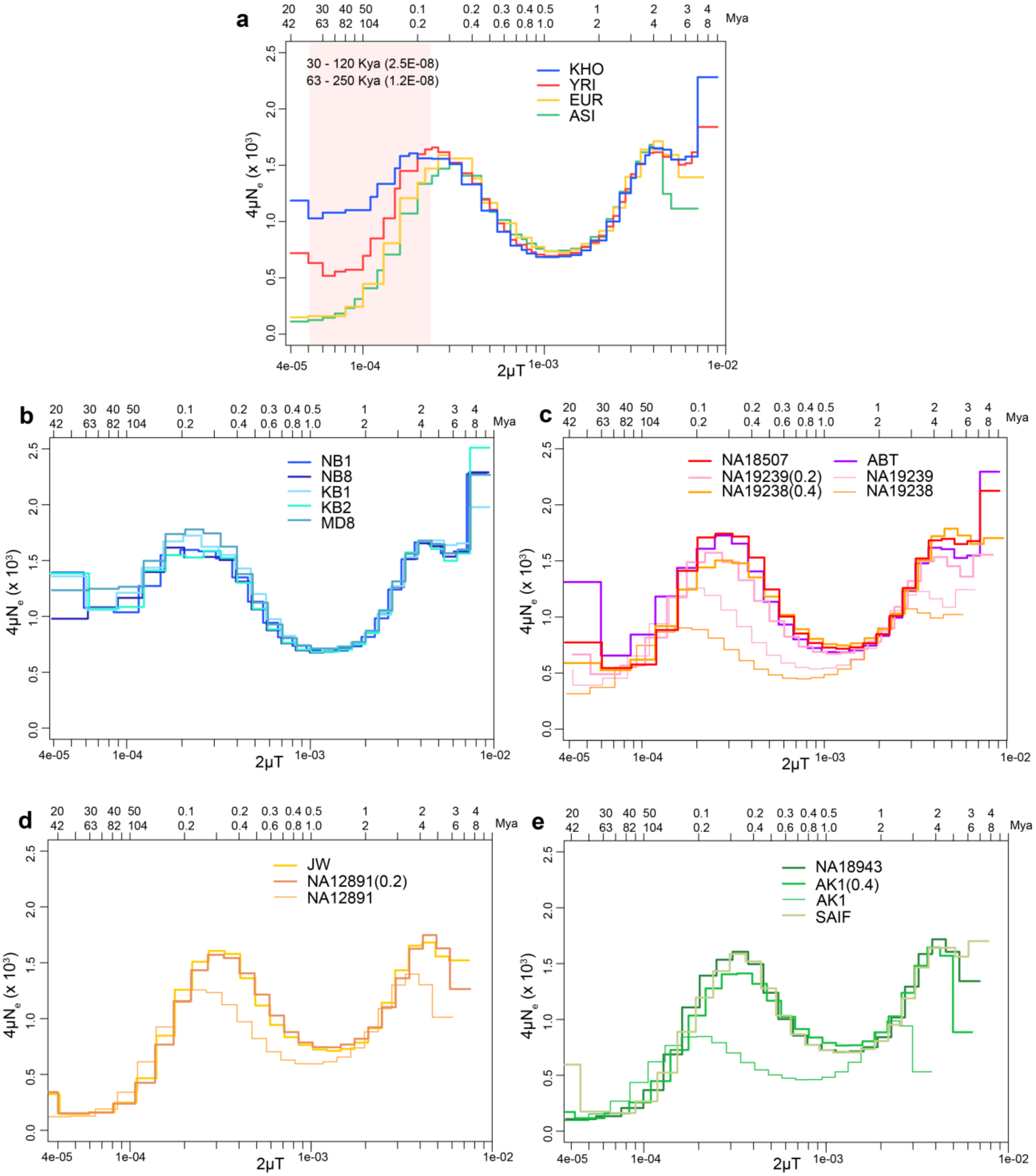

Supplementary Figure 13. Time scaling by mutation rates.
The PSMC estimates are illustrated as same as Fig. 3 in the main text. Here we showed the effect of the parameter, mutation rate, on the time scale in the plot. We used 2.5e-8/site/gen in the Fig. 3, as shown in the upper X axis in this figure. As well as, the time scale used 1.2e-8/site/gen is also shown in the upper X axis. The smaller time is scaled using the higher mutation rate, 2.5e-8/site/gen. This time scale becomes to be almost two times when the slower mutation rate, 1.2e-8, is used.

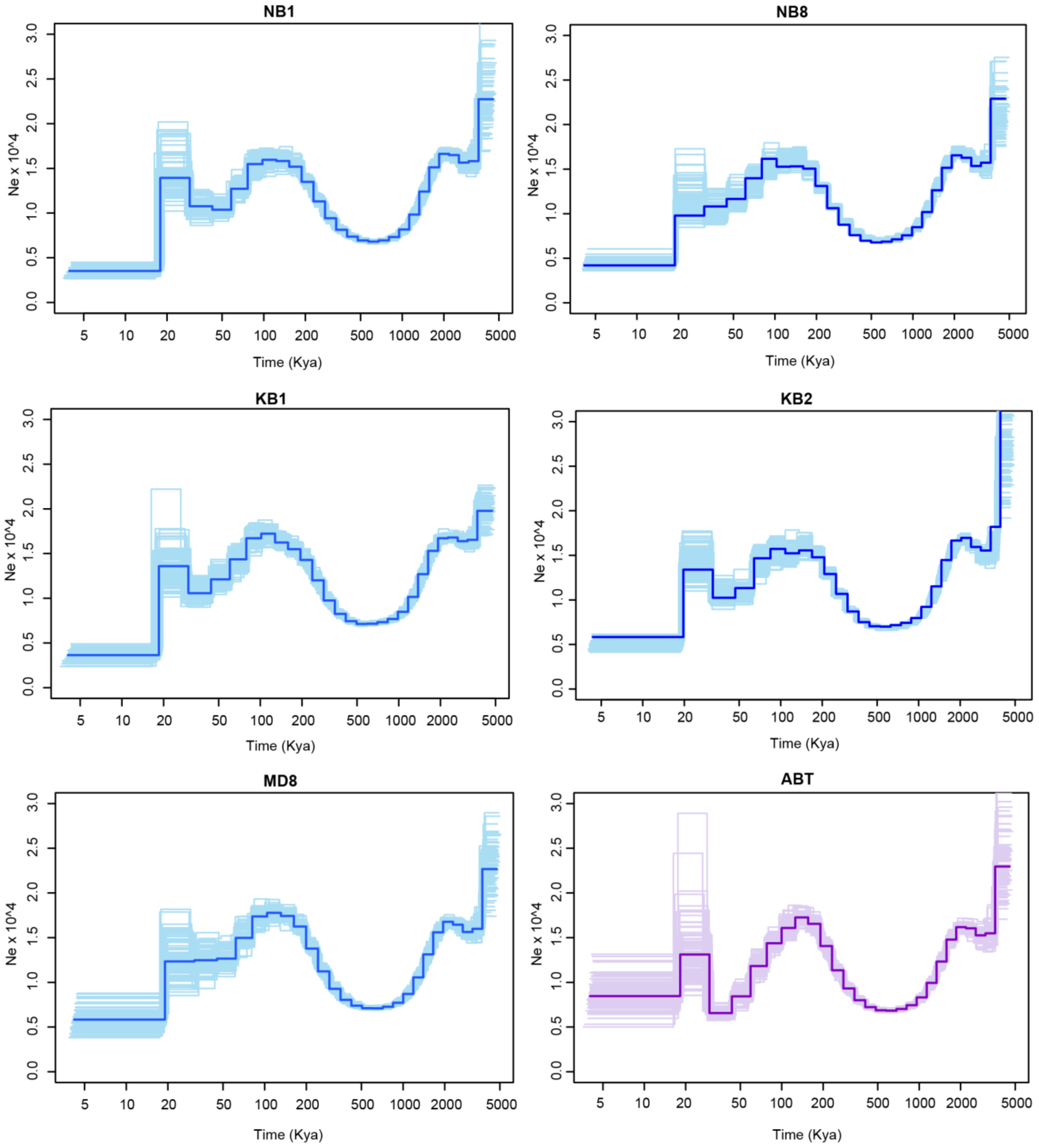

Supplementary Figure 14. The bootstrap test of the PSMC analysis. The X and Y axis indicates time (kya) and effective population size ($\times 10^4$), respectively. Both are scheduled based on the 2.5e-08 mutation rate/site/generation. The bootstrap test was performed using the option in the PSMC package. Each graph shows results of 100 repeats.

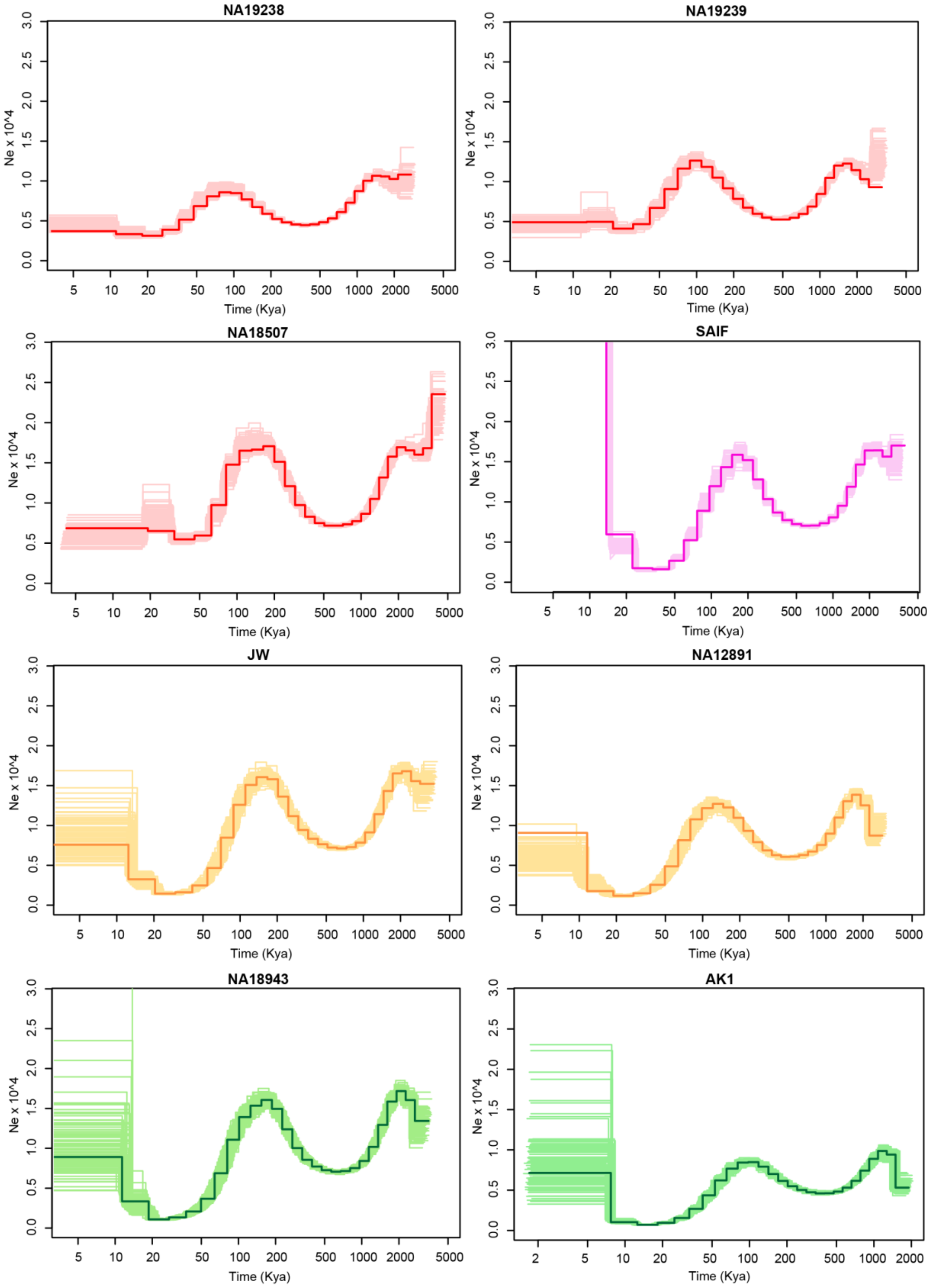

Supplementary Figure 14 (continued).

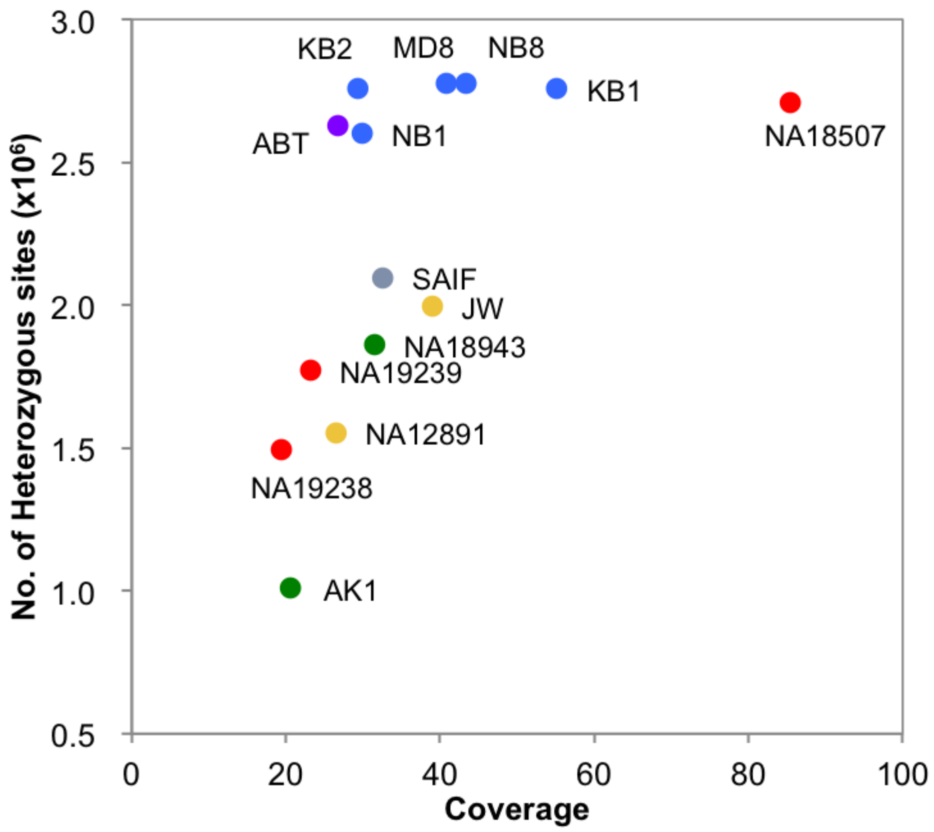

Supplementary Figure 15. Associations of sequencing coverage and number of heterozygotes in the 14 genome sequence datasets.

The X axis indicates the sequencing coverage of each individual genome, and the Y axis indicates the number of heterozygotes identified in the variants calling of each individual. The high coverage supposes a resulting high number of heterozygotes. The ABT (27-fold coverage) genome has a number of heterozygotes similar to NA18507, which was sequenced to the deepest coverage. The sequence coverage of the ABT genome might be high enough to identify heterozygotes.

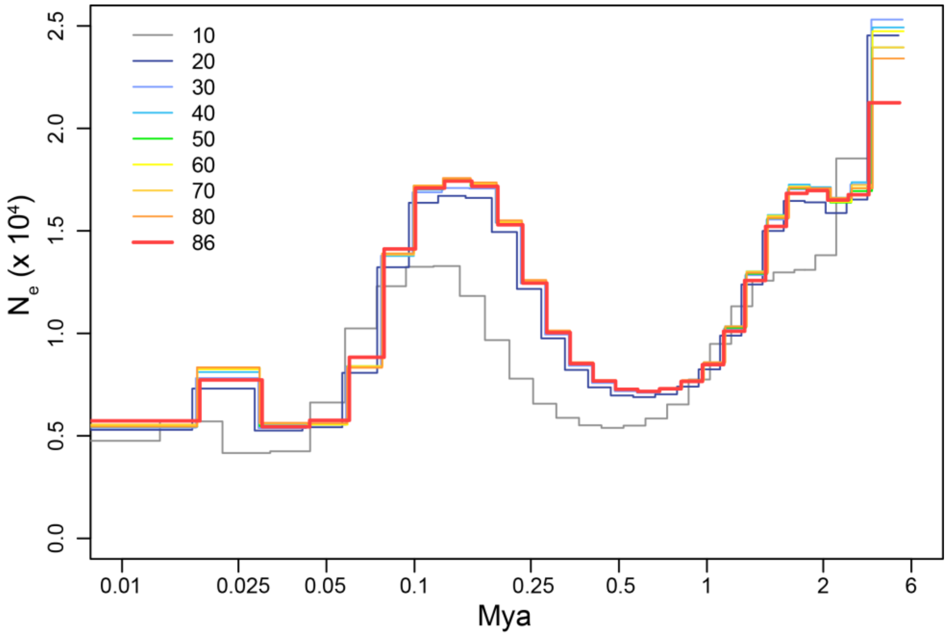

Supplementary Figure 16. The effect of sequencing quality on the PSMC inference for NA18507.

Each color indicates a different sequencing coverage as shown in the left top of the graph.

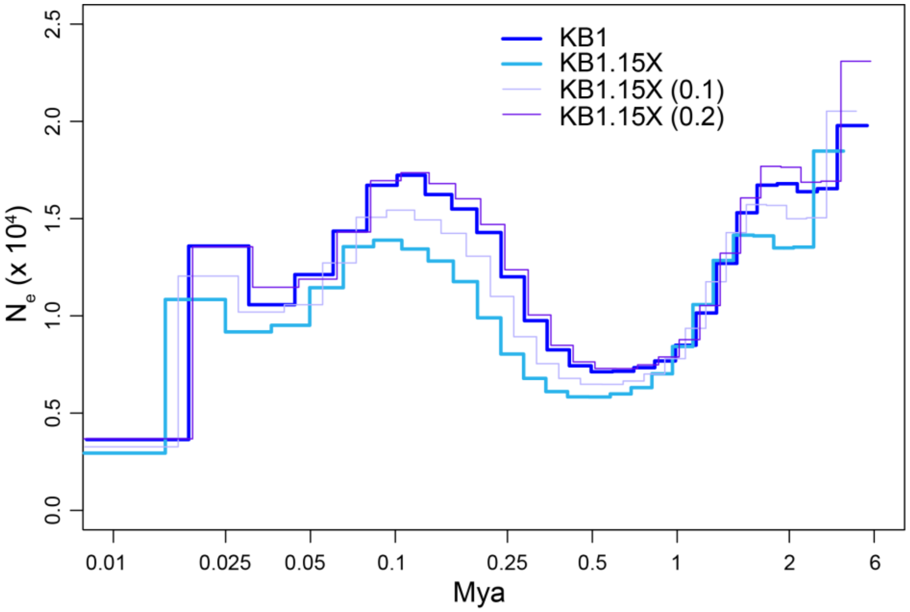

Supplementary Figure 17. The FNR correction of PSMC estimates using the KB1 genome dataset.

Each color represents the different FNR options. The PSMC estimates of the 15-fold sequencing coverage KB1 genome can be corrected to the estimates of the original coverage KB1 genome.

Supplementary Table 1. The SNP genotype datasets of 1,462 individuals. The bold letters represent Khoisan populations.

	HapMap	HGDP	Schlebusch et al 2012	14 whole genomes	Total	
No. SNPs	1,505,108	660,765	2,293,320	-	419,969	
Individuals	243	1,041	164	14	1,462	
Populations	3	39	8	9	48	
		**San**	**Ju/'hoansi**	**NB1, NB8**	**Ju/'hoansi, San**	**22**
			**!Xun**	**MD8**	**!Xun**	**20**
			**≠Khomani**		**≠Khomani**	**39**
			**Nama**		**Nama**	**20**
			**Karretjie**		**Karretjie**	**20**
			**Khwe**		**Khwe**	**17**
			**/Gui and //Gana**		**/Gui and //Gana**	**15**
				**KB1, KB2**	**Tuu-speaker**	**2**
		Biaka_Pygmies			Biaka_Pygmies	31
		Mbuti_Pygmies			Mbuti_Pygmies	15
		Bantu_NE			Bantu_NE	12
		Bantu_SE	Bantu_SE	ABT	Bantu_SE	26
		Bantu_SW			Bantu_SW	3
	YRI	Yoruba		NA18507, NA19238, NA19239	Yoruba	113
		Mandenka			Mandenka	24
		Mozabite			Mozabite	30
		Palestinian			Palestinian	51
		Bedouin			Bedouin	48
		Druze			Druze	47
	CEU	French, North_Italian, Tuscan, Orcadian		JW, NA12891	European	144
		French_Basque			French_Basque	24
		Sardinian			Sardinian	28
		Russian			Russian	25
		Adygei			Adygei	17
		Makrani			Makrani	25
		Brahui			Brahui	25
		Balochi			Balochi	25
		Pathan			Pathan	23
		Kalash			Kalash	25
		Sindhi			Sindhi	25
		Burusho			Burusho	25
				SAIF	Indian	1
		Hazara			Hazara	24
		Uygur			Uygur	10
		Colombians			Colombians	13
		Maya			Maya	25
		Pima			Pima	25
		Karitiana			Karitiana	24
		NAN_Melanesian			NAN_Melanesian	19
		Papuan			Papuan	17
		Surui			Surui	21
		Yakut			Yakut	25
		Cambodians			Cambodians	11
		Japanese		NA18943	Japanese	30
				AK1	Korean	1
		Daur, Mongola, Tu			Mongols	29
		Dai, Miaozu, Lahu, Hezhen, Naxi, Oroqen, She, Tujia, Xibo, Yizu			Chinese ethnic groups	97
	CHB	Han			Han Chinese	124

Supplementary Table 2. Summary of admixture analyses

	KHO	YRI	CEU	ASI	UD	Total
**ADMIXTURE**	K=4					
**NB1**	1.000	0.000	0.000	0.000	-	1
**NB8**	1.000	0.000	0.000	0.000	-	1
**KB1**	0.951	0.049	0.000	0.000	-	1
**KB2**	0.872	0.128	0.000	0.000	-	1
**MD8**	0.840	0.148	0.012	0.000	-	1
**ABT**	0.221	0.779	0.000	0.000	-	1
**PCAdmix**	Window=40SNPs					
**NB1**	0.981	0.001	0.001	-	0.016	1
**NB8**	0.983	0.003	0.000	-	0.014	1
**KB1**	0.957	0.007	0.001	-	0.036	1
**KB2**	0.910	0.036	0.000	-	0.053	1
**MD8**	0.917	0.023	0.002	-	0.059	1
**ABT**	0.145	0.705	0.003	-	0.147	1
**HAPMIX**	Lambda=5 / Theta=0.001~0.5					
**NB1**	0.985	0.012	0.003	-	0.000	1
**NB8**	0.983	0.016	0.001	-	0.000	1
**KB1**	0.914	0.083	0.002	-	0.001	1
**KB2**	0.844	0.144	0.005	-	0.007	1
**MD8**	0.863	0.130	0.005	-	0.003	1
**ABT**	0.211	0.786	0.000	-	0.002	1
**dpmix**	Switch penalty=10					
**NB1**	0.950	0.029	0.021	-	-	1
**NB8**	0.953	0.029	0.017	-	-	1
**KB1**	0.899	0.082	0.019	-	-	1
**KB2**	0.835	0.140	0.025	-	-	1
**MD8**	0.831	0.139	0.030	-	-	1
**ABT**	0.236	0.741	0.023	-	-	1
**Consistent results**						
**NB1**	0.903	0.000	0.000	-	0.096	1
**NB8**	0.907	0.000	0.000	-	0.093	1
**KB1**	0.781	0.006	0.000	-	0.213	1
**KB2**	0.666	0.024	0.000	-	0.310	1
**MD8**	0.669	0.012	0.000	-	0.319	1
**ABT**	0.061	0.464	0.000	-	0.475	1

Supplementary Table 3. References for Figure 4

Figure 3 symbol captions	References
Rainfall decrease (Stadial)<br>Rainfall increase (Stadial)	Broccoli et al. 2006 (52), Mulitza et al. 2008 (53), Carto et al. 2009 (28), Chiang et al. 2013 (54)
Rainfall decrease (Glacial) ~25-115, ~130-180 kya<br>Rainfall increase (Glacial) ~25-115, ~130-180 kya	deMenocal et al. 1993 (22), Broccoli et al. 2006 (52), Braconnot et al. 2007 (55)
Moisture increase (Glacial) ~25-115, ~130-180 kya	Stuut et al. 2002 (24), Chase et al. 2007 (23)
Dry (Precession variability) ~95-85 kya	Pokras and Mix 1987 (56), deMenocal et al. 1993 (22)
Moist (Precession variability) ~95-85 kya	Parridge et al. 1997 (27)
Dry (Glacial and Precession variability) ~95-85 kya	Weldeab et al. 2007 (26)
Moist ~87-100 kya	Ziegler et al. 2013 (29)
Dry (Stadials)<br>Moist (Stadials)<br>Small changes (Stadials)	Stager et al. 2011 (57), Ziegler et al. 2013 (29)

Supplementary References

1. de Filippo, C., Bostoen, K., Stoneking, M. & Pakendorf, B. Bringing together linguistic and genetic evidence to test the Bantu expansion. *Proc. Biol. Sci.* **279,** 3256–3263 (2012).

2. Biesele, Megan and Royal-/o/oo, K. *The Ju/'hoansi of Botswana and Namibia. In The Cambridge Ecnyclopedia of Hunters and Gatherers, in The Cambridge Ecnyclopedia of Hunters and Gatherers*. 205–209 (Cambridge University Press, 1999).

3. Tishkoff, S. A. *et al.* The genetic structure and history of Africans and African Americans. *Science.* **324,** 1035–1044 (2009).

4. Henn, B. M. *et al.* Hunter-gatherer genomic diversity suggests a southern African origin for modern humans. *Proc. Natl. Acad. Sci. U. S. A.* **108,** 5154–5162 (2011).

5. Schlebusch, C. M. *et al.* Genomic variation in seven Khoe-San groups reveals adaptation and complex African history. *Science.* **338,** 374–379 (2012).

6. Semino, O., Santachiara-Benerecetti, A. S., Falaschi, F., Cavalli-Sforza, L. L. & Underhill, P. A. Ethiopians and Khoisan share the deepest clades of the human Y-chromosome phylogeny. *Am. J. Hum. Genet.* **70,** 265–268 (2002).

7. Gonder, M. K., Mortensen, H. M., Reed, F. A., de Sousa, A. & Tishkoff, S. A. Whole-mtDNA genome sequence analysis of ancient African lineages. *Mol. Biol. Evol.* **24,** 757–768 (2007).

8. Petersen, D. C. *et al.* Complex patterns of genomic admixture within southern Africa. *PLoS Genet.* **9,** e1003309 (2013).

9. Pickrell, J. K. *et al.* Ancient west Eurasian ancestry in southern and eastern Africa. *Proc. Natl. Acad. Sci.* (2014).

10. Schuster, S. C. *et al.* Complete Khoisan and Bantu genomes from southern Africa. *Nature* **463,** 943–947 (2010).

11. Alexander, D. H., Novembre, J. & Lange, K. Fast model-based estimation of ancestry in unrelated individuals. *Genome Res.* **19,** 1655–1664 (2009).

12. Patterson, N., Price, A. L. & Reich, D. Population structure and eigenanalysis. *PLoS Genet.* **2,** e190 (2006).

13. Loh, P.-R. *et al.* Inferring admixture histories of human populations using linkage disequilibrium. *Genetics* **193,** 1233–54 (2013).

14. Li, J. Z. *et al.* Worldwide human relationships inferred from genome-wide patterns of variation. *Science.* **319,** 1100–4 (2008).

15. Pickrell, J. K. *et al.* The genetic prehistory of southern Africa. *Nat. Commun.* **3,** 1143 (2012).

16. Veeramah, K. R. *et al.* An early divergence of KhoeSan ancestors from those of other modern humans is supported by an ABC-based analysis of autosomal resequencing data. *Mol. evobiologylution* **29,** 617–630 (2012).

17. Li, H. & Durbin, R. Inference of human population history from individual whole-genome sequences. *Nature* **475,** 493–496 (2011).

18. Nachman, M. W. & Crowell, S. L. Estimate of the mutation rate per nucleotide in humans. *Genetics* **156,** 297–304 (2000).

19. Gronau, I., Hubisz, M. J., Gulko, B., Danko, C. G. & Siepel, A. Bayesian inference of ancient human demography from individual genome sequences. *Nat. Genet.* **43,** 1031–1034 (2011).

20. Mellars, P. Why did modern human populations disperse from Africa ca. 60,000 years ago? A new model. *Proc. Natl. Acad. Sci. USA* **103,** 9381–9386 (2006).

21. Barker, P. & Gasse, F. New evidence for a reduced water balance in East Africa during the Last Glacial Maximum: implication for model-data comparison. *Quat. Sci. Rev.* **22,** 823–837 (2003).

22. deMenocal, P. B., Ruddiman, W. F. & Pokras, E. M. Influences of high- and low-latitude processes on African terrestrial climate: Pleistocene eolian records from equatorial atlantic ocean drilling program site 663. *Paleoceanography* **8,** 209–242 (1993).

23. Chase, B. M. & Meadows, M. E. Late Quaternary dynamics of southern Africa's winter rainfall zone. *Earth-Science Rev.* **84,** 103–138 (2007).

24. Stuut, J.-B. W. *et al.* A 300-kyr record of aridity and wind strength in southwestern Africa: inferences from grain-size distributions of sediments on Walvis Ridge, SE Atlantic. *Mar. Geol.* **180,** 221–233 (2002).

25. Prell, W. L. & Kutzbach, J. E. Monsoon variability over the past 150,000 years. *J. Geophys. Res.* **92,** 8411–8425 (1987).

26. Weldeab, S., Lea, D. W., Schneider, R. R. & Andersen, N. 155,000 years of West African monsoon and ocean thermal evolution. *Science.* **316,** 1303–1307 (2007).

27. Partridge, T. C., deMenocal, P. B., Lorentz, S. A., Paiker, M. J. & Vogel, J. C. Orbital forcing of climate over south Africa: a 200,000-year rainfall record from the pretoria saltpan. *Quat. Sci. Rev.* **16,** 1125–1133 (1997).

28. Carto, S. L., Weaver, A. J., Hetherington, R., Lam, Y. & Wiebe, E. C. Out of Africa and into an ice age: on the role of global climate change in the late Pleistocene migration of early modern humans out of Africa. *J. Hum. Evol.* **56,** 139–51 (2009).

29. Ziegler, M. *et al.* Development of Middle Stone Age innovation linked to rapid climate change. *Nat. Commun.* **4,** 1905 (2013).

30. Campbell, C. D. *et al.* Estimating the human mutation rate using autozygosity in a founder population. *Nat. Genet.* **44,** 1277–2381 (2012).

31. Conrad, D. F. *et al.* Variation in genome-wide mutation rates within and between human families. *Nat. Genet.* **43,** 712–714 (2011).

32. Kong, A. *et al.* Rate of de novo mutations and the importance of father's age to disease risk. *Nature* **488,** 471–475 (2012).

33. Li, H. & Durbin, R. Fast and accurate short read alignment with Burrows-Wheeler transform. *Bioinformatics* **25,** 1754–1760 (2009).

34. McKenna, A. *et al.* The Genome Analysis Toolkit: a MapReduce framework for analyzing next-generation DNA sequencing data. *Genome Res.* **20,** 1297–1303 (2010).

35. Picard, http://picard.sourceforge.net

36. Lander, E. S. & Waterman, M. S. Genomic mapping by fingerprinting random clones: a mathematical analysis. *Genomics* **2,** 231–239 (1988).

37. The International HapMap Consortium. International HapMap project. *Nature* **426,** 789–795 (2003).

38. CEPH, http://www.cephb.fr/en/hgdp

39. Purcell, S. *et al.* PLINK: a tool set for whole-genome association and population-based linkage analyses. *Am. J. Hum. Genet.* **81,** 559–575 (2007).

40. Goecks, J., Nekrutenko. A, Taylor, J., and Galaxy Team. Galaxy: a comprehensive approach for supporting accessible, reproducible, and transparent computational research in the life sciences. Genome Biol. **25**, R86 (2010).

41. Price, A. L. *et al.* Sensitive detection of chromosomal segments of distinct ancestry in admixed populations. *PLoS Genet.* **5,** e1000519 (2009).

42. Brisbin, A. G. Linkage Analysis for categorical traits and ancestry assignment in admixed individuals. (2010).

43. Bedoya-Reina, O. C. *et al.* Galaxy tools to study genome diversity. *Gigascience* **2,** 17 (2013).

44. http://bochet.gcc.biostat.washington.edu/beagle/1000_Genomes.phase1_release_v3/

45. Browning, S. R. & Browning, B. L. Rapid and accurate haplotype phasing and missing-data inference for whole-genome association studies by use of localized haplotype clustering. *Am. J. Hum. Genet.* **81,** 1084–1097 (2007).

46. Hudson, R. R. Generating samples under a Wright-Fisher neutral model of genetic variation. *Bioinformatics* **18,** 337-338 (2002).

47. Prado-Martinez, J. *et al.* Great ape genetic diversity and population history. *Nature* **499,** 471–475 (2013).

48. Rambaut A, Drummond AJ. Tracer v1.5.0. Available from http://beast.bio.ed.ac.uk/Tracer (2009).

49. Patterson, N., Richter, D. J., Gnerre, S., Lander, E. S. & Reich, D. Genetic evidence for complex speciation of humans and chimpanzees. *Nature* **441,** 1103–1108 (2006).

50. Tamura, K. *et al.* MEGA5: molecular evolutionary genetics analysis using maximum likelihood, evolutionary distance, and maximum parsimony methods. *Mol. Biol. Evol.* **28,** 2731–2739 (2011).

51. Kloss-Brandstätter, A. *et al.* HaploGrep: a fast and reliable algorithm for automatic classification of mitochondrial DNA haplogroups. *Hum Mutat* **32,** 25–32 (2011).

52. Broccoli, A. J., Dahl, K. A. & Stouffer, R. J. Response of the ITCZ to Northern Hemisphere cooling. *Geophys. Res. Lett.* **33,** L01702 (2006).

53. Mulitza, S. *et al.* Sahel megadroughts triggered by glacial slowdowns of Atlantic meridional overturning. *Paleoceanography* **23,** 1–11 (2008).

54. Chiang, J. C. H., Biasutti, M. & Battisti, D. S. Sensitivity of the Atlantic Intertropical Convergence Zone to Last Glacial Maximum boundary conditions. *Paleoceanography* **18,** 1–17 (2003).

55. Braconnot, P. *et al.* Results of PMIP2 coupled simulations of the Mid-Holocene and Last Glacial Maximum – Part 1: experiments and large-scale features. *Clim. Past* **3,** 261–277 (2007).

56. Pokras, E. M. & Mix, A. C. Earth's precession cycle and quaternary climatic change in tropical Africa. *Nature* **326,** 486–487 (1987).

57. Stager, J. C., Ryves, D. B., Chase, B. M. & Pausata, F. S. R. Catastrophic drought in the Afro-Asian monsoon region during Heinrich event 1. *Science.* **331,** 1299–1302 (2011).
